# HLA-B*13 :01 Is a Predictive Marker of Dapsone-Induced Severe Cutaneous Adverse Reactions in Thai Patients

**DOI:** 10.3389/fimmu.2021.661135

**Published:** 2021-05-04

**Authors:** Patompong Satapornpong, Jirawat Pratoomwun, Pawinee Rerknimitr, Jettanong Klaewsongkram, Nontaya Nakkam, Thanyada Rungrotmongkol, Parinya Konyoung, Niwat Saksit, Ajanee Mahakkanukrauh, Warayuwadee Amornpinyo, Usanee Khunarkornsiri, Therdpong Tempark, Kittipong Wantavornprasert, Pimonpan Jinda, Napatrupron Koomdee, Thawinee Jantararoungtong, Ticha Rerkpattanapipat, Chuang-Wei Wang, Dean Naisbitt, Wichittra Tassaneeyakul, Manasalak Ariyachaipanich, Thapana Roonghiranwat, Munir Pirmohamed, Wen-Hung Chung, Chonlaphat Sukasem

**Affiliations:** ^1^ Division of Pharmacogenomics and Personalized Medicine, Department of Pathology, Faculty of Medicine Ramathibodi Hospital, Mahidol University, Bangkok, Thailand; ^2^ Laboratory for Pharmacogenomics, Somdech Phra Debaratana Medical Center (SDMC), Ramathibodi Hospital, Bangkok, Thailand; ^3^ Division of General Pharmacy Practice, Department of Pharmaceutical Care, College of Pharmacy, Rangsit University, Pathum Thani, Thailand; ^4^ Department of Clinical Chemistry, Faculty of Medical Technology, Huachiew Chalermprakiet University, Samut Prakan, Thailand; ^5^ The Skin and Allergy Research Unit, Chulalongkorn University, Bangkok, Thailand; ^6^ Division of Dermatology, Department of Medicine, Faculty of Medicine, Chulalongkorn University, Bangkok, Thailand; ^7^ Division of Allergy and Clinical Immunology, Department of Medicine, Faculty of Medicine, Chulalongkorn University, Bangkok, Thailand; ^8^ King Chulalongkorn Memorial Hospital, Thai Red Cross Society, Bangkok, Thailand; ^9^ Department of Pharmacology, Faculty of Medicine, Khon Kaen University, Khon Kaen, Thailand; ^10^ Biocatalyst and Environmental Biotechnology Research Unit, Department of Biochemistry, Faculty of Science, Chulalongkorn University, Bangkok, Thailand; ^11^ Program in Bioinformatics and Computational Biology, Graduated School, Chulalongkorn University, Bangkok, Thailand; ^12^ Pharmacy Unit, Udon Thani Hospital, Udon Thani, Thailand; ^13^ Unit of Excellence on Pharmacogenomic Pharmacokinetic and Pharmacotherapeutic Researches (UPPER), School of Pharmaceutical Sciences, University of Phayao, Phayao, Thailand; ^14^ Department of Medicine, Faculty of Medicine, Khon Kaen University, Khon Kaen, Thailand; ^15^ Division of Dermatology, Department of Internal Medicine, Khon Kaen Hospital, Khon Kaen, Thailand; ^16^ Division of Dermatology, Department of Pediatrics, Faculty of Medicine, Chulalongkorn University, Bangkok, Thailand; ^17^ Division of Allergy Immunology and Rheumatology, Department of Medicine, Faculty of Medicine, Ramathibodi Hospital, Mahidol University, Bangkok, Thailand; ^18^ Department of Dermatology, Drug Hypersensitivity Clinical and Research Center, Chang Gung Memorial Hospital (CGMH), Taipei, Taiwan; ^19^ Cancer Vaccine and Immune Cell Therapy Core Laboratory, Chang Gung Memorial Hospital, Linkou, Taiwan; ^20^ Department of Dermatology, Xiamen Chang Gung Hospital, Xiamen, China; ^21^ Department of Molecular and Clinical Pharmacology, MRC Centre for Drug Safety Science, University of Liverpool, Liverpool, United Kingdom; ^22^ Skin Center, Ruampaet Dr.ANAN Hospital, Surin, Thailand; ^23^ Department of Pediatrics, Prapokklao Hospital, Chantaburi, Thailand; ^24^ Whole-Genome Research Core Laboratory of Human Diseases, Chang Gung Memorial Hospital, Keelung, Taiwan; ^25^ Genomic Medicine Core Laboratory, Chang Gung Memorial Hospital, Linkou, Taiwan; ^26^ The Thai Severe Cutaneous Adverse Drug Reaction (THAI-SCAR) Research Group, Bangkok, Thailand

**Keywords:** dapsone-induced severe cutaneous adverse reactions, *HLA* class I and II alleles, HLA-B*13:01, cytochrome P450, Thais and Taiwaneses

## Abstract

HLA-B*13:01 allele has been identified as the genetic determinant of dapsone hypersensitivity syndrome (DHS) among leprosy and non-leprosy patients in several studies. Dapsone hydroxylamine (DDS-NHOH), an active metabolite of dapsone, has been believed to be responsible for DHS. However, studies have not highlighted the importance of other genetic polymorphisms in dapsone-induced severe cutaneous adverse reactions (SCAR). We investigated the association of *HLA* alleles and cytochrome P450 (CYP) alleles with dapsone-induced SCAR in Thai non-leprosy patients. A prospective cohort study, 16 Thai patients of dapsone-induced SCARs (5 SJS-TEN and 11 DRESS) and 9 Taiwanese patients of dapsone-induced SCARs (2 SJS-TEN and 7 DRESS), 40 dapsone-tolerant controls, and 470 general Thai population were enrolled. *HLA* class I and II alleles were genotyped using polymerase chain reaction-sequence specific oligonucleotides (PCR-SSOs). *CYP2C9*, *CYP2C19*, and *CYP3A4* genotypes were determined by the TaqMan real-time PCR assay. We performed computational analyses of dapsone and DDS-NHOH interacting with HLA-B*13:01 and HLA-B*13:02 alleles by the molecular docking approach. Among all the *HLA* alleles, only HLA-B*13:01 allele was found to be significantly associated with dapsone-induced SCARs (OR = 39.00, 95% CI = 7.67–198.21, p = 5.3447 × 10^−7^), SJS-TEN (OR = 36.00, 95% CI = 3.19–405.89, p = 2.1657 × 10^−3^), and DRESS (OR = 40.50, 95% CI = 6.38–257.03, p = 1.0784 × 10^−5^) as compared to dapsone-tolerant controls. Also, HLA-B*13:01 allele was strongly associated with dapsone-induced SCARs in Asians (OR = 36.00, 95% CI = 8.67–149.52, p = 2.8068 × 10^−7^) and Taiwanese (OR = 31.50, 95% CI = 4.80–206.56, p = 2.5519 × 10^−3^). Furthermore, dapsone and DDS-NHOH fit within the extra-deep sub pocket of the antigen-binding site of the HLA-B*13:01 allele and change the antigen-recognition site. However, there was no significant association between genetic polymorphism of cytochrome P450 (*CYP2C9*, *CYP2C19*, and *CYP3A4*) and dapsone-induced SCARs (SJS-TEN and DRESS). The results of this study support the specific genotyping of the HLA-B*13:01 allele to avoid dapsone-induced SCARs including SJS-TEN and DRESS before initiating dapsone therapy in the Asian population.

## Introduction

Dapsone (4, 4’-diaminodiphenylsulfone, DDS) is wildly used for treatment of infection and inflammation including of leprosy, *Pneumocystis jiroveci* pneumonia (PJP), or *Toxoplasma gondii* encephalitis in human immunodeficiency virus (HIV) prophylaxis, neutrophilic dermatoses, dermatitis herpetiformis, and autoimmune bullous disease (1). However, the most frequent adverse drug reactions of dapsone are dose-dependent adverse effects (hemolytic anemia and methemoglobinemia) and rarely dose-independent adverse effects (dapsone hypersensitivity syndrome) (2). Dapsone hypersensitivity syndrome (DHS) or dapsone-induced hypersensitivity reactions (DIHRs) is a life-threatening drug reaction and usually manifested between the 4 and 6 weeks after initiation of treatment. The clinically characterized through fever, rash, hepatitis or systemic involvement, lymphadenopathy, and abnormal hematologic system (eosinophilia or atypical lymphocytosis) (3). This entity is also termed DHS and DIHRs has been considered a manifestation of drug reaction with eosinophilia and systemic symptoms (DRESS). There was found approximately 0.5–3.6% of patients treated with dapsone have been reported to develop DHS and the mortality rate of 9.9% (4). Especially, about 2% of leprosy patients treated with dapsone have a DHS and 12.5% of mortality (5, 6). According to data from the King Chulalongkorn Memorial Hospital, Thailand reported during 2004–2014, dapsone is the 5^th^ ranked common culprit drug causing DRESS in Thai patients (7).

Severe cutaneous adverse drug reactions (SCARs) is a type of adverse drug reactions (ADRs) that remains a rare but potentially severe life-threatening adverse effect and major problems for both clinical treatment and pharmaceutical industry (8). SCARs comprise a heterogeneous groups of distinct clinical manifestation, including of Stevens-Johnson syndrome (SJS), toxic epidermal necrolysis (TEN), drug reaction with eosinophilia and systemic symptoms (DRESS), and acute generalized exanthematous pustulosis (AGEP) (9). Clinical characteristic of SJS, SJS/TEN overlap, and TEN are acute and rapid progression of mucous detachment and systemic symptoms. They are differentiated by the severe of skin detachment, involving <10% of body surface area (BSA) in SJS, 10–30% of BSA in SJS/TEN overlap, and >30% of BSA in TEN (10). According to the RegiSCARs study, SJS has a mortality rates in the range from about 10% and more than 40% for TEN (11). The main causes of SJS-TEN are medicines and risk factors such as HIV infection, renal disease, liver disease, and active systemic autoimmune disease (12). Drug reaction with eosinophilia and systemic symptoms (DRESS) are characterized by a skin rash usually occurring more than 2 weeks after drug initiation with fever, hepatitis or internal organ involvement, lymphadenopathy, and hematological abnormalities (eosinophilia or atypical lymphocytosis) (13). The mortality rate of DRESS is approximately 10% (14).

Although the exact mechanism of SCARs remains unclear, numerous studies have described the associations between *human leukocyte antigen* (*HLA*) and cytochrome P450 genes with the specific drug hypersensitivity reaction (15, 16). For example, HLA-B*15:02 with carbamazepine-induced SJS/TEN is recommended for Han Chinese, Malaysia, India, and Thailand (17–20). On the contrary, HLA-A*31:01 is the main genetic determinant for carbamazepine-induced SJS, TEN, and DRESS in Japanese and Europeans (21, 22). Thus, HLA-B*15:02 is phenotype-specific with carbamazepine-induced SJS/TEN in each population. Additionally, there were important discovered the drug metabolism enzymes of phenytoin-induced SJS-TEN. The metabolize processes of phenytoin to p-HPPH (inactive form), arene oxides were cause of phenytoin hypersensitivity reactions by poor metabolizer (PM) alleles of mutation CYP2C9 gene consist of CYP2C9*2 and CYP2C9*3 in Asian (23, 24).

In previous studies, only HLA-B*13:01 was strongly associated with DHS in leprosy Han Chinese (odds ratio 122.1, *p*-value = 6.038 × 10^−12^ and odds ratio 20.53, *p*-value = 6.84 × 10^−25^) and dapsone-induced DRESS in non-leprosy Thais (odds ratio = 60.75, *p*-value = 0.0001) (25–27). Furthermore, Dapsone is metabolized through acetylation and N-hydroxylation. In human study, they found a relation between the rate of N-hydroxylation and clearance of dapsone by cytochrome P450 (28). Genetic polymorphisms of CYP2C9, CYP2C19, and CYP3A4 influenced the dapsone metabolism and cause of DHS through by DDS-NHOH (dapsone hydroxylamine) (29). Nevertheless, there are no data describing whether *HLA class I*, *II* alleles and cytochrome P450 is a valid marker for prediction of dapsone-induced SCARs in non-leprosy patients in addition to HLA-B*13:01. Consequently, the aim of this study was to investigate the contributing pharmacogenetics markers association between *HLA class I*, *II*, cytochrome P450, and dapsone-induced SCARs in Thai non-leprosy patients.

## Materials and Methods

### Subjects

We enrolled 16 non-leprosy Thai patients with dapsone-induced SCARs consist of 5 SJS-TEN patients and 11 DRESS patients were classified by RegiSCAR criteria. SJS is defined as skin detachment less than 10% of BSA, SJS/TEN overlap has 10–30% of BSA involved, and TEN as skin detachment more than 30% of BSA (30). Moreover, SJS-TEN with severe ocular surface complications (SOC) was diagnosis with history of acute-onset high fever, serious mucocutaneous illness with skin eruption, and the involvement of at least two mucosal sites (oral cavity and ocular surface) (31). DRESS was defined by the triad of skin eruption, hematological involvement, and internal organ involvements according to the RegiSCAR Group Diagnosis Score (13). All patients with dapsone-induced SCARs were accessed through review of photographs, pathologic slides, and medical records by two dermatologists. Furthermore, there were two cases with SJS-TEN and seven cases with DRESS in the Taiwan population. Forty dapsone-tolerant controls who had been non-leprosy Thai patients and received dapsone more than 6 months without any cutaneous adverse reaction.

All of participants in this study from the Faculty of Medicine Ramathibodi Hospital, Mahidol University, Faculty of Medicine, Chulalongkorn University; Faculty of Medicine, Khon Kaen University; Udon Thani Hospital and the Thai Severe Cutaneous Adverse Drug Reaction (THAI-SCAR) research group. In addition, 470 unrelated healthy Thai population were recruited for this study. The study was approved by the ethics committee of Ramathibodi Hospital (MURA2016/105), Khon Kaen University (HE510837) and Udon Thani Hospital (22/2563). Written informed consent was obtained from each patients before enrollment.

There were collected the clinical data of dapsone-induced SCARs and controls consist of age, gender, indication for dapsone treatment, dapsone dose (mg/day), co-medication, complete blood cell count (CBC), blood urea nitrogen (BUN), serum creatinine (SCr), aspartate aminotransferase (AST) or serum glutamic oxaloacetic transaminase (SGOT), and alanine aminotransferase (ALT) or serum glutamic pyruvic transaminase (SGPT).

### *HLA* Class I and II Genotyping

*HLA* class I and II alleles were genotyped using sequence-specific oligonucleotides (PCR-SSOs). Diluted DNA sample was amplified polymerase chain reaction (PCR) by GeneAmp^®^PCR System 9700 (Applied Biosystems, Waltham, USA). The PCR product was then hybridized against a panel of oligonucleotide probes on coated polystyrene microspheres that had sequences complementary to stretches of polymorphism within the target *HLA class I* and *II* alleles using the Lifecodes HLA SSO typing kits (Immucor, West Avenue, Stamford, USA) and detection by the Luminex^®^IS 100 system (Luminex Corporation, Austin, TX, USA). *HLA class I* and *II* alleles were performed using MATCH IT DNA software version 3.2.1 (One Lambda, Canoga Park, CA, USA).

### *CYP2C9*, *2C19*, and *3A4* Genotyping

The genotyping of candidate genes [CYP2C9*2 (430C > T, rs1799853), CYP2C9*3 (1075A > C, rs1057910), CYP2C19*2 (681G > A, rs4244285), CYP2C19*3 (636G > A, rs4986893), CYP2C19*17 (-806C > T, rs12248560), CYP3A4*1B (c.-392A > G, rs2740574), and CYP3A4*18 (c.878T > C, rs28371759)] were genotyped by the TaqMan real time PCR assay (ABI, Foster City, CA, USA). The SNPs genotyping will be conducted using the real-time PCR ViiA7 (ABI, Foster City, CA, USA).

### *In Silico* Model of Dapsone, DDS-NHOH, and HLA-B*13:01 Complex

The 3D structures of HLA-B*13:01 and HLA-B*13:02 were modeled by using HLA-B*5201 from Protein Data Bank (3W39.PDB) as the template structure. The protonation states of all ionizable amino acids were assigned at pH 7.0 using PROPKA 3.0 (32). The structural geometries of Dapsone and DDS-NHOH were generated and fully optimized by the HF/6-31 G(d) level of theory using Gaussian09 program (33). Then, each drug was docked into the binding pocket of specific HLA with 100 independent docking runs using the CDOCKER module implemented in Discovery Studio 2.5 (Accelrys, Inc.).

### Statistical Analysis

Chi-square test and Fisher’s exact test were used to analyze the association between dapsone-induced SCARs, dapsone controls, and healthy Thai population. Statistical analysis was performed using SPSS version 16.0 (SPSS Inc., Chicago, IL, USA). The association was estimated by calculating the odds ratio (OR) with a 95% confidence interval (CI). Sensitivity, specificity, positive predictive value (PPV), and negative predictive value (NPV) were calculated. The corrected *P*-values (*P_c_*) for the multiple comparison of *HLA* alleles (16 for *HLA-A*, 22 for *HLA-B*, 20 for *HLA-C*, 18 for *HLA-DRB1*, 9 for *HLA-DQA1*, and 11 for *HLA-DQB1*) were calculated using Bonferroni’s correction. *P*-values were less than 0.05 (two-tailed) was considered to indicate statistically significant.

## Results

### Clinical Characteristic

The demographic and clinical data of patients with dapsone-induced SCARs and controls are listed in **Table 1**. Patients who were diagnosed with SJS, TEN, and DRESS were validated as “probable” and “definite” case by dermatologists using RegiSCAR criteria and all of dapsone-induced SJS-TEN patients without severe ocular complications (SOC). The 16 patients with dapsone-induced SCARs consisted of 10 females (62.5%) and 6 males (37.5%), with a median age of 45 (range 2.5–64) years. Meanwhile, 28 (70%) dapsone controls were females with a median age of 41.5 (range 4–75) years. The median onset time of SJS-TEN and DRESS was 32.5 (14–56) and 31.5 (3–63) days, respectively, after exposure to dapsone. The median onset time of SJS-TEN and DRESS were not significantly different. Dapsone was used among the cases and controls for the HIV prophylaxis (25.00% of cases, 17.50% of controls), systemic lupus erythematosus (SLE) (18.75% of cases, 22.50% of controls), chronic bullous disease of childhood (CBDC) (6.25% of cases, 7.50% of controls), and immune thrombocytopenic purpura (ITP) (18.75% of cases, 2.50% of controls). Eight patients (20.00%) had a previous history of cotrimoxazole-induced hypersensitivity reaction in the dapsone-tolerant group. Dapsone dosages used were 100 mg/day, while two patients (2.5 and 4 years old) received 18 and 16.7 mg/day, respectively. The hematological abnormalities and hepatitis were more prominent among the dapsone cases, as shown in **Table 1**. Furthermore, the most common of co-medication used among the dapsone cases and controls were colchicine, efavirenz, lamivudine, and acyclovir.

**Table 1:** Demographic and clinical data between dapsone-induced SCARs and tolerant controls.

Clinical characteristic	Dapsone-induced SCARs (n = 16)	Dapsone controls (n = 40)	*p*-value
**Sex n (%)**			
- Male	6 (37.5)	12 (30)	0.5872
- Female	10 (62.5)	28 (70)	
**Age (range) years**			
Median	45 (2.5–64)*	41.5 (4-75)	0.6783
**Indication for medication n (%)**			
**Autoimmune disease**			
SLE	3 (18.75)	9 (22.50)	1.0000
MCTD	1 (6.25)	0	0.2857
ITP	3 (18.75)	1 (2.50)	0.0659
**Autoimmune bullous disease**			
CBDC	1 (6.25)	3 (7.50)	1.0000
Dermatitis herpetiformis	1 (6.25)	0	0.2857
Pemphigus foliaceus	0	1 (2.50)	1.0000
Pemphigus vulgaris	0	1 (2.50)	1.0000
Bullous pemphigoid	0	4 (10.00)	0.3148
**Prophylaxis**			
HIV	4 (25.00)	7 (17.50)	0.7108
**Other**			
Eosinophilic cellulitis	1 (6.25)	0	0.2857
Dyshidrosis	1 (6.25)	0	0.2857
Folliculitis decalvans	1 (6.25)	0	0.2857
**Type of SCARs n (%)**			
- SJS/TEN	5 (31.25)	–	–
- DRESS	11 (68.75)		
**Onset of duration: SCARs [median (range)] day**			
- SJS/TEN	32.5 (14–56)	> 60	–
- DRESS	31.5 (3–63)		
**Dapsone dose (mg/day)**	18**, 100	16.7**, 100	0.9037
**Co-medication**			
Colchicine	4 (25.00)	5 (12.50)	0.2586
Efavarez	3 (18.75)	4 (10.00)	0.3947
Lamivudine	2 (12.50)	4 (10.00)	1.0000
Hydroxychloroquine	2 (12.50)	3 (7.50)	0.6172
Fluconazole	1 (6.25)	3 (7.50)	1.0000
Acyclovir	0	5 (12.50)	0.3068
**History of ADRs n (%)**			
cloxacillin	1 (6.25)	0	0.2857
aspirin	1 (6.25)	0	0.2857
co-trimoxazole	0	8 (20.00)	0.0892
penicillin	0	2 (5.00)	1.0000
sulfasalazine	0	1 (2.50)	1.0000
**Clinical laboratory [median (range)]**			
Hematocrit (%)	33 (14.1–42)	36 (9.8–46)	0.0667
Hemoglobin (g/dl)	10.1 (4.9–14)	11.95 (8.8–30.7)	**0.0126**
White blood cell (cell/mm^3^)	11,400 (3,600–48,980)	8,350 (3,330–16,830)	0.3262
AST (U/L)	122 (20–2,013)	28 (8–189)	**1.4057 × 10^−4^**
ALT (U/L)	204 (47–945)	26 (6–159)	**1.3939 × 10^−5^**
BUN (mg/dl)	10.25 (8–24)	11 (7–24)	0.7211
SCr (mg/dl)	0.69 (0.45–1.32)	0.65 (0.21–1.64)	0.5733

*Age at the development of dapsone-induced hypersensitivity; **1.5 mg/kg/day for pediatric dose; ALT, alanine Aminotransferase; AST, aspartate amino transferase; BUN, blood urea nitrogen; SCr, serum creatinine; SCARs, severe cutaneous adverse reactions; SJS, Stevens-Johnson syndrome; TEN, toxic epidermal necrolysis; DRESS, drug reaction with eosinophilia and systemic symptoms; CBDC, chronic bullous disease of childhood; HIV, Human Immunodeficiency Virus; ITP, Immune Thrombocytopenic Purpura; MCTD, Mixed connective tissue disease; SLE, Systemic Lupus Erythematosus; Significant different p-value <0.05. In bold: Data analysis result was presented statistical significance (p-value < 0.05).

### Association Between Dapsone-Induced SCARs and *HLA* Class I, II Alleles

The association between *HLA* class I and II alleles and dapsone-induced SCARs were evaluated by comparing the SCARs group with the dapsone-tolerant controls group and the Thai general population. The number of HLA-B*13:01 carriers were 13 of 16 (81.25%) in dapsone-induced SCARs, 4 of 40 (10.00%) in dapsone-tolerant controls, and 54 of 470 (11.49%) in Thai population. The frequency of HLA-B*13:01 was significantly associated with dapsone-induced SCARs when compared with dapsone controls (OR: 39.00; 95% CI: 7.67–198.21 and *p*-value = 5.3447 × 10^−7^) and general Thai population (OR: 33.38; 95% CI: 9.22–120.91 and *p*-value = 8.8033 × 10^−10^) as shown in **Table 2**. Also, other *HLA* alleles were significant association with dapsone-induced SCARs including of HLA-A*24:07, HLA-C*03:04, HLA-DRB1*15:01, and HLA-DQB1*06:01 by *p*-value = 0.0494, 0.0023, 0.0258, and 0.0258, respectively (**Table 2**). In this study, HLA-B*15:02 was not significantly associated with dapsone-induced SCARs (*p*-value = 0.1005). The HLA-B*13:01-C*03:04, HLA-B*13:01-DRB1*15:01, HLA-B*13:01-DQB1*06:01, and HLA-DRB1*15:01-DQB1*06:01 haplotypes showed significant association when compared between dapsone-induced SCARs and tolerant controls (**Table 2**).

**Table 2:** Association of *HLA* class I and II alleles with dapsone-induced SCARs.

Pharmacogenomics markers	Dapsone-induced SCARs (n = 16)	Dapsone controls (n = 40)	Thai population (n = 470)	Dapsone-induced SCARs cases *versus* controls			Dapsone-induced SCARs cases *versus* Thais		
				Odds ratio (95% CI)	*P*-value	*P*c-value	Odds ratio (95% CI)	*P*-value	*P*c-value
***HLA class I***									
HLA-A*02:01	0	6 (15.00%)	51 (10.85%)	0.16 (0.01–3.03)	0.1676	NS	0.25 (0.02–4.18)	0.3949	NS
HLA-A*02:03	1 (6.25%)	8 (20.00%)	99 (21.06%)	0.27 (0.03–2.33)	0.4210	NS	0.25 (0.03–1.91)	0.2128	NS
HLA-A*02:06	0	3 (7.50%)	21 (4.47%)	0.32 (0.02–6.65)	0.5498	NS	0.63 (0.04–10.92)	1.0000	NS
HLA-A*02:07	2 (12.50%)	9 (22.50%)	68 (14.47%)	0.49 (0.09–2.58)	0.4829	NS	0.85 (0.19–3.79)	1.0000	NS
HLA-A*11:01	10 (62.50%)	16 (40.00%)	211 (44.89%)	2.50 (0.76–8.25)	0.1272	NS	2.05 (0.73–5.72)	0.1643	NS
HLA-A*24:02	4 (25.00%)	5 (12.50%)	95 (20.21%)	2.33 (0.54–10.14)	0.2586	NS	1.32 (0.42–4.17)	0.7512	NS
HLA-A*24:07	**4 (25.00%)**	**2 (5.00%)**	**39 (8.30%)**	**6.33 (1.03–38.98)**	**0.0494**	0.7896	**3.68 (1.13–11.97)**	**0.0441**	0.7057
HLA-A*30:01	1 (6.25%)	3 (7.50%)	20 (4.26%)	0.82 (0.08–8.55)	1.0000	NS	1.50 (0.19–11.93)	0.5123	NS
HLA-A*33:01	0	3 (7.50%)	3 (0.64%)	0.32 (0.02–6.65)	0.5498	NS	4.05 (0.20–81.59)	1.0000	NS
HLA-A*33:03	**1 (6.25%)**	**13 (32.50%)**	99 (21.06%)	**0.14 (0.02–1.17)**	**0.0471**	0.7537	0.25 (0.03–1.91)	0.2128	NS
HLA-B*07:05	1 (6.25%)	1 (2.50%)	24 (5.11%)	2.60 (0.15–44.28)	0.4935	NS	1.24 (0.16–9.77)	0.5763	NS
HLA-B*13:01	**13 (81.25%)**	**4 (10.00%)**	**54 (11.49%)**	**39.00 (7.67–198.21)**	**5.3447 × 10^−7^**	**1.1758 × 10^−5^**	**33.38 (9.22–120.91)**	**8.8033 × 10^−10^**	**1.9367 × 10^−8^**
HLA-B*13:02	1 (6.25%)	3 (7.50%)	20 (4.26%)	0.82 (0.08–8.55)	1.0000	NS	1.50 (0.19–11.93)	0.5123	NS
HLA-B*15:02	5 (31.25%)	4 (10.00%)	71 (15.11%)	4.09 (0.93–17.94)	0.1005	NS	2.55 (0.86–7.57)	0.0879	NS
HLA-B*15:35	1 (6.25%)	1 (2.50%)	3 (0.64%)	2.60 (0.15–44.28)	0.4935	NS	10.38 (1.02–105.69)	0.1257	NS
HLA-B*18:01	1 (6.25%)	5 (12.50%)	36 (7.66%)	0.47 (0.05–4.34)	0.6622	NS	0.80 (0.10–6.26)	1.0000	NS
HLA-B*27:06	1 (6.25%)	2 (5.00%)	12 (2.55%)	1.27 (0.11–15.03)	1.0000	NS	2.54 (0.31–20.86)	0.3564	NS
HLA-B*38:02	0	4 (10.00%)	39 (8.30%)	0.25 (0.01–4.84)	0.3148	NS	0.33 (0.02–5.62)	0.6294	NS
HLA-B*40:01	1 (6.25%)	7 (17.50%)	58 (12.34%)	0.31 (0.04–2.79)	0.4163	NS	0.47 (0.06–3.65)	0.7062	NS
HLA-B*44:03	0	5 (12.50%)	42 (8.94%)	0.19 (0.01–3.75)	0.3068	NS	0.31 (0.02–5.18)	0.3823	NS
HLA-B*46:01	2 (12.50%)	10 (25.00%)	122 (25.96%)	0.43 (0.08–2.22)	0.4751	NS	0.41 (0.09–1.82)	0.3802	NS
HLA-B*51:01	0	3 (7.50%)	40 (8.51%)	0.33 (0.02–6.65)	0.5498	NS	0.32 (0.02–5.47)	0.3840	NS
HLA-B*58:01	1 (6.25%)	5 (12.50%)	57 (12.13%)	0.47 (0.05–4.34)	0.6622	NS	0.48 (0.06–3.73)	0.7065	NS
HLA-C*01:02	2 (12.50%)	10 (25.00%)	143 (30.43%)	0.43 (0.08–2.22)	0.4751	NS	0.33 (0.07–1.46)	0.1669	NS
HLA-C*03:02	1 (6.25%)	7 (17.50%)	69 (14.68%)	0.31 (0.04–2.79)	0.4163	NS	0.39 (0.05–2.98)	0.4885	NS
HLA-C*03:04	**8 (50.00%)**	**4 (10.00%)**	**66 (14.04%)**	**9.00 (2.17–37.38)**	**0.0023**	**0.0464**	**6.12 (2.22–16.87)**	**9.3405 × 10^−4^**	**1.8681 × 10^−2^**
HLA-C*03:09	**2 (12.50%)**	1 (2.50%)	**1 (0.21%)**	5.57 (0.47–66.33)	0.1934	NS	**67.00 (5.73–783.13)**	**0.0030**	0.0599
HLA-C*04:01	1 (6.25%)	2 (5.00%)	44 (9.36%)	1.27 (0.11–15.03)	1.0000	NS	0.65 (0.08–5.00)	1.0000	NS
HLA-C*06:02	1 (6.25%)	4 (10.00%)	40 (8.51%)	0.60 (0.06–5.82)	1.0000	NS	0.72 (0.09–5.57)	1.0000	NS
HLA-C*07:01	0	7 (17.50%)	58 (12.34%)	0.14 (0.01–2.52)	0.1740	NS	0.21 (0.01–3.61)	0.2375	NS
HLA-C*07:02	1 (6.25%)	10 (25.00%)	101 (21.49%)	0.20 (0.02–1.71)	0.1499	NS	0.24 (0.03–1.87)	0.2124	NS
HLA-C*07:04	2 (12.50%)	6 (15.00%)	46 (9.79%)	0.81 (0.15–4.51)	1.0000	NS	1.32 (0.29–5.98)	0.6655	NS
HLA-C*08:01	6 (37.50%)	7 (17.50%)	90 (19.15%)	2.83 (0.77–10.38)	0.1610	NS	2.53 (0.89–7.15)	0.1021	NS
***HLA class II***									
HLA-DRB1*03:01	1 (6.25%)	6 (15.00%)	43 (9.15%)	0.38 (0.04–3.42)	0.6595	NS	0.66 (0.09–5.13)	1.0000	NS
HLA-DRB1*04:05	2 (12.50%)	1 (2.50%)	45 (9.57%)	5.57 (0.47–66.33)	0.1934	NS	1.35 (0.29–6.13)	0.6609	NS
HLA-DRB1*07:01	1 (6.25%)	9 (22.50%)	83 (17.66%)	0.23 (0.03–1.98)	0.2514	NS	0.31 (0.04–2.39)	0.3280	NS
HLA-DRB1*08:03	2 (12.50%)	1 (2.50%)	14 (2.98%)	5.57 (0.47–66.33)	0.1934	NS	4.65 (0.96–22.46)	0.0932	NS
HLA-DRB1*09:01	2 (12.50%)	2 (5.00%)	88 (18.72%)	2.71 (0.35–21.16)	0.5696	NS	0.62 (0.14–2.78)	0.7476	NS
HLA-DRB1*11:01	2 (12.50%)	1 (2.50%)	15 (3.19%)	5.57 (0.47–66.33)	0.1934	NS	4.33 (0.90–20.79)	0.1036	NS
HLA-DRB1*12:02	2 (12.50%)	8 (20.00%)	134 (28.51%)	0.57 (0.11–3.04)	0.7068	NS	0.36 (0.08–1.59)	0.2560	NS
HLA-DRB1*14:01	2 (12.50%)	8 (20.00%)	51 (10.85%)	0.57 (0.11–3.04)	0.7068	NS	1.17 (0.26–5.31)	0.6900	NS
HLA-DRB1*15:01	**7 (43.75%)**	**5 (12.50%)**	**72 (15.32%)**	**5.44 (1.39–21.24)**	**0.0258**	0.4645	**4.29 (1.55–11.91)**	**0.0077**	0.1385
HLA-DRB1*15:02	2 (12.50%)	14 (35.00%)	124 (26.38%)	0.27 (0.05–1.34)	0.1135	NS	0.39 (0.09–1.78)	0.2608	NS
HLA-DRB1*16:02	2 (12.50%)	9 (22.50%)	52 (11.06%)	0.49 (0.09–2.58)	0.4829	NS	1.15 (0.25–5.19)	0.6951	NS
HLA-DQA1*01:01	5 (31.25%)	22 (55.00%)	196 (41.70%)	0.37 (0.11–1.27)	0.1081	0.9728	0.64 (0.22–1.86)	0.4038	NS
HLA-DQA1*01:02	8 (50.00%)	18 (45.00%)	183 (38.94%)	1.22 (0.38–3.90)	0.7347	NS	1.57 (0.58–4.25)	0.3729	NS
HLA-DQA1*01:03	2 (12.50%)	3 (7.50%)	34 (7.23%)	1.76 (0.27–11.69)	0.6172	NS	1.83 (0.40–8.39)	0.3352	NS
HLA-DQA1*02:01	0	6 (15.00%)	81 (17.23%)	0.16 (0.01–3.03)	0.1676	NS	0.15 (0.01–2.44)	0.0866	0.7797
HLA-DQA1*03:01	1 (6.25%)	1 (2.50%)	40 (8.51%)	2.60 (0.15–44.28)	0.4935	NS	0.72 (0.09–5.57)	1.0000	NS
HLA-DQA1*03:02	3 (18.75%)	4 (10.00%)	125 (26.60%)	2.08 (0.41–10.56)	0.3947	NS	0.64 (0.18–2.27)	0.5781	NS
HLA-DQA1*05:01	1 (6.25%)	7 (17.50%)	49 (10.43%)	0.31 (0.04–2.79)	0.4163	NS	0.57 (0.07–4.43)	1.0000	NS
HLA-DQA1*05:05	**4 (25.00%)**	3 (7.50%)	**31 (6.60%)**	4.11 (0.80–21.03)	0.0937	NS	**4.72 (1.44–15.49)**	**0.0221**	0.1987
HLA-DQA1*06:01	2 (12.50%)	7 (17.50%)	107 (22.77%)	0.67 (0.12–3.65)	1.0000	NS	0.49 (0.11–2.17)	0.5420	NS
HLA-DQB1*02:01	1 (6.25%)	6 (15.00%)	47 (10.00%)	0.38 (0.04–3.42)	0.6595	NS	0.60 (0.08–4.65)	1.0000	NS
HLA-DQB1*02:02	1 (6.25%)	8 (20.00%)	68 (14.47%)	0.27 (0.03–2.33)	0.4210	NS	0.39 (0.05–3.03)	0.7124	NS
HLA-DQB1*03:01	5 (31.25%)	11 (27.50%)	151 (32.13%)	1.19 (0.34–4.24)	0.7559	NS	0.96 (0.33–2.81)	0.9411	NS
HLA-DQB1*03:02	2 (12.50%)	2 (5.00%)	37 (7.87%)	2.71 (0.35–21.16)	0.5696	NS	1.67 (0.37–7.64)	0.3729	NS
HLA-DQB1*03:03	2 (12.50%)	3 (7.50%)	101 (21.49%)	1.76 (0.27–11.69)	0.6172	NS	0.52 (0.12–2.33)	0.5413	NS
HLA-DQB1*04:01	1 (6.25%)	1 (2.50%)	35 (7.45%)	2.60 (0.15–44.28)	0.4935	NS	0.83 (0.11–6.46)	1.0000	NS
HLA-DQB1*05:01	2 (12.50%)	10 (25.00%)	121 (25.74%)	0.43 (0.08–2.22)	0.4751	NS	0.41 (0.09–1.84)	0.3795	NS
HLA-DQB1*05:02	6 (37.5%)	19 (47.50%)	182 (38.72%)	0.66 (0.20–2.17)	0.4965	5.4613	0.95 (0.34–2.66)	0.9213	10.1342
HLA-DQB1*05:03	1 (6.25%)	6 (15.00%)	37 (7.87%)	0.38 (0.04–3.42)	0.6595	NS	0.78 (0.10–6.07)	1.0000	NS
HLA-DQB1*06:01	**7 (43.75%)**	**5 (12.50%)**	**63 (13.40%)**	**5.44 (1.39–21.24)**	**0.0258**	0.2839	**5.03 (1.81–13.97)**	**0.0038**	**0.0413**
HLA-DQB1*06:02	1 (6.25%)	1 (2.50%)	14 (2.98%)	2.60 (0.15–44.28)	0.4935	NS	2.17 (0.27–17.61)	0.3993	NS
**Haplotype**									
HLA-B*13:01/ C*03:04	**8 (50.00%)**	**2 (5.00%)**	**31 (6.60%)**	**19.00 (3.38–106.84)**	**0.0003**	**0.0124**	**14.16 (4.98–40.29)**	**6.7468 × 10^−6^**	**0.0003**
HLA-B*13:01/ DRB1*15:01	**5 (31.25%)**	**2 (5.00%)**	**12 (2.55%)**	**8.64 (1.47–50.79)**	**0.0161**	0.6449	**17.35 (5.21–57.74)**	**9.6792 × 10^−5^**	**0.0039**
HLA-B*13:01/ DQB1*06:01	**5 (31.25%)**	**1 (2.50%)**	**9 (1.91%)**	**17.73 (1.87–168.00)**	**0.0056**	0.1857	**23.28 (6.69–80.95)**	**3.3199 × 10^−5^**	**0.0011**
HLA-B*13:01/ DRB1*15:01/ DQB1*06:01	**3 (18.75%)**	1 (2.50%)	**7 (1.49%)**	9.00 (0.86–94.24)	0.0659	NS	**15.26 (3.54–65.76)**	**0.0031**	0.1563
HLA-DRB1*15:01/ DQB1*06:01	**5 (31.25%)**	**3 (7.50%)**	**33 (7.02%)**	**5.61 (1.15–27.26)**	**0.0351**	NS	**6.02 (1.97–18.35)**	**0.0052**	0.1504

Significant different P-value <0.05; HLA-A, human leucocyte antigen-A; HLA-B, human leucocyte antigen-B; HLA-C, human leucocyte antigen-C; HLA-DRB1, human leukocyte antigen-DRB1; HLA-DQA1, human leucocyte antigen-DQA1; HLA-DQB1, human leucocyte antigen-DQB1; SCARs, severe cutaneous adverse reactions; OR, odds ratio; 95% CI, 95% Confidence Interval; P-value, probability value were calculated using Fisher’s exact test or Chi-square test; Pc-value, Corrected p-value were adjusted by Bonferroni’s correction (16 for HLA-A, 22 for HLA-B, 20 for HLA-C, 18 for HLA-DRB1, 9 for HLA-DQA1, and 11 for HLA-DQB1); NS, Not significant. In bold: Data analysis result was presented statistical significance (p-value < 0.05).

When *p*-values were adjusted by Bonferroni’s correction (16 for *HLA-A*, 22 for *HLA-B*, 20 for *HLA-C*, 18 for *HLA-DRB1*, 9 for *HLA-DQA1*, and 11 for *HLA-DQB1*), only HLA-B*13:01 allele was strongly associated in dapsone-induced SCARs when compared with tolerant controls and general Thai population. Also, HLA-B*13:01–C*03:04 haplotype was significantly associated with dapsone-induced SCARs with corrected *p*-value = 0.0124. The sensitivity, specificity, PPV, and NPV of HLA–B*13:01 allele for prediction of dapsone-induced SCARs were 76.47, 92.31, 12.37, and 99.64%, respectively (**Table 3**). We then examined the carrier rate of HLA-B*13:01 and HLA-C*03:04 alleles among the study population (Thais and Taiwanese) with dapsone-induced SCARs. We found HLA-B*13:01 was significantly associated with dapsone-induced SCARs when compared with dapsone controls (OR: 36.00; 95% CI: 8.67–149.52 and *P*c-value = 2.8068 × 10^−7^) and with general Thai population (OR: 30.82; 95% CI:11.11–85.47 and *P*c-value = 1.7827 × 10^−12^) (**Table 4**). Furthermore, there was a statistical significance between HLA-C*03:04 and dapsone-induced SCARs in Asian patients.

**Table 3:** Sensitivity, Specificity, PPV, and NPV between dapsone-induced SCARs and tolerant control.

*HLA* allele	Dapsone-induced SCARs				Dapsone-induced SJS/TEN					Dapsone-induced DRESS			
	Sensitivity	Specificity	PPV	NPV	Sensitivity		Specificity	PPV	NPV	Sensitivity	Specificity	PPV	NPV
HLA-B*13:01	76.47	92.31	12.37	99.64	50.00		97.30	20.80	99.28	69.23	94.74	15.74	99.54
HLA-C*03:04	66.67	81.82	4.95	99.42	42.86		94.74	10.36	99.15	55.56	85.71	5.23	99.27
HLA-DRB1*15:01	58.33	79.55	3.89	99.26	37.50		94.59	8.97	99.07	44.44	83.33	3.65	99.06
HLA-DQB1*06:01	58.33	79.55	3.89	99.26	28.57		92.11	4.89	98.91	50.00	85.37	4.63	99.18
HLA-B*13:01/-C*03:04	80.00	82.61	6.13	99.66	60.00		95.00	14.56	99.41	71.43	86.36	6.92	99.53
HLA-B*13:01/-DRB1*15:01	71.43	77.55	4.32	99.48	50.00		92.68	8.84	99.24	60.00	82.61	4.67	99.32
HLA-B*13:01/-DQB1*06:01	83.33	78.00	5.10	99.70	50.00		90.70	7.09	99.22	80.00	84.78	6.95	99.67
HLA-B*13:01/ DRB1*15:01/ DQB1*06:01	75.00	75.00	4.09	99.53	50.00		90.70	7.09	99.22	66.67	81.25	4.81	99.42
HLA-DRB1*15:01/-DQB1*06:01	62.50	77.08	3.73	99.31	40.00		92.50	7.04	99.09	50.00	82.22	3.84	99.14

HLA-B, human leucocyte antigen-B; HLA-C, human leucocyte antigen-C; HLA-DRB1, human leukocyte antigen-DRB1; HLA-DQB1, human leucocyte antigen-DQB1; SCARs, severe cutaneous adverse reactions; SJS, Stevens-Johnson syndrome; TEN, toxic epidermal necrolysis; DRESS, drug reaction with eosinophilia and systemic symptoms; PPV, positive predictive value; NPV, negative predictive value; the prevalence of dapsone hypersensitivity syndrome was 1.4%^2^.

**Table 4:** Association between HLA-B*13:01/HLA-C*03:04 and dapsone-induced SCARs in Asians.

Populations	Type	Total (n)	HLA-B*13:01 carrier n (%)	Odds ratio (95% CI)	*P*-value	*P*c-value
Asians	SCARs	25	20 (80.00%)	**36.00 (8.67–149.52)**	**1.2758 × 10^−8^**	**2.8068 × 10^−7^**
				**30.82 (11.11–85.47)**	**8.1032 × 10^−14^**	**1.7827 × 10^−12^**
	SJS-TEN	7	6 (85.71%)	**54.00 (5.12–569.39)**	**1.2545 × 10^−4^**	**2.7599 × 10^−3^**
				**46.22 (5.46–391.26)**	**1.9936 × 10^−5^**	**4.3858 × 10^−4^**
	DRESS	18	14 (77.78%)	**31.50 (6.91–143.62)**	**2.4458 × 10^−7^**	**5.3809 × 10^−6^**
				**26.96 (8.57–84.88)**	**5.8209 × 10^−10^**	**1.2806 × 10^−8^**
Taiwanese	SCARs	9	7 (77.78%)	**31.50 (4.80–206.56)**	**1.1599 × 10^−4^**	**2.5519 × 10^−3^**
				**26.96 (5.46–133.13)**	**1.1578 × 10^−5^**	**2.5472 × 10^−4^**
	SJS-TEN	2	2 (100.00%)	40.56 (1.67–985.44)	**0.0174**	0.3833
				38.21 (1.81–806.45)	**0.0139**	0.3048
	DRESS	7	5 (71.43%)	**22.50 (3.24–156.27)**	**0.0015**	**0.0321**
				**19.26 (3.65–101.71)**	**4.2707 × 10^−4^**	**9.3954 × 10^−3^**
Thais	SCARs	16	13 (81.25%)	**39.00 (7.67–198.21)**	**5.3447 × 10^−7^**	**1.1758 × 10^−5^**
				**33.38 (9.22–120.91)**	**8.8033 × 10^−10^**	**1.9367 × 10^−8^**
	SJS-TEN	5	4 (80.00%)	**36.00 (3.19–405.89)**	**2.1657 × 10^−3^**	**4.7645 × 10^−2^**
				**30.82 (3.38–280.78)**	**9.1996 × 10^−4^**	**2.0239 × 10^−2^**
	DRESS	11	9 (81.82%)	**40.50 (6.38–257.03)**	**1.0784 × 10^−5^**	**2.3725 × 10^−4^**
				**34.67 (7.29–164.67)**	**2.9734 × 10^−7^**	**6.5415 × 10^−6^**
	Tolerant group	40	4 (10.00%)	–	–	–
	General Thai population	470	54 (11.49%)	–	–	–
**Populations**	**Type**	**Total (n)**	HLA-C*03:04 carrier n (%)	**Odds ratio (95% CI)**	***P*-value**	***P*c-value**
Asians	SCARs	24	11 (45.83%)	**7.62 (2.06–28.18)**	**0.0011**	**0.0210**
				**5.18 (2.23–12.05)**	**3.0309 × 10^−4^**	**6.0619 × 10^−3^**
	SJS-TEN	7	4 (57.14%)	12.00 (1.95–73.97)	**0.0108**	0.2170
				8.16 (1.79–37.29)	**0.0106**	0.2115
	DRESS	17	7 (41.18%)	6.30 (1.53–25.91)	**0.0110**	0.2208
				4.29 (1.58–11.65)	**0.0071**	0.1430
Taiwanese	SCARs	8	3 (37.50%)	5.40 (0.92–31.55)	0.0795	1.5902
				3.67 (0.86–15.73)	0.0943	1.8852
	SJS-TEN	2	1 (50.00%)	9.00 (0.47–173.34)	0.2265	4.5296
				6.12 (0.38–99.06)	0.2640	5.2801
	DRESS	6	2 (33.33%)	4.50 (0.62–32.82)	0.1687	3.3744
				3.06 (0.55–17.04)	0.2062	4.1231
Thais	SCARs	16	8 (50.00%)	**9.00 (2.17–37.38)**	**0.0023**	**0.0464**
				**6.12 (2.22–16.87)**	**9.3405 × 10^−4^**	**1.8681 × 10^−2^**
	SJS-TEN	5	3 (60.00%)	**13.50 (1.71–106.56)**	**0.0212**	0.4249
				**9.18 (1.51–55.99)**	**0.0237**	0.4734
	DRESS	11	5 (45.45%)	**7.50 (1.56–36.17)**	**0.0155**	0.3093
				**5.10 (1.51–17.19)**	**0.0138**	0.2752
	Tolerant group	40	4 (10.00%)	–	–	–
	General Thai population	470	66 (14.04%)	–	–	–

Significant different P-value <0.05; HLA-B, human leucocyte antigen-B; HLA-C, human leucocyte antigen-C; SCARs, Severe cutaneous adverse reactions; SJS, Stevens-Johnson syndrome; TEN, toxic epidermal necrolysis; DRESS, drug reaction with eosinophilia and systemic symptoms; OR, odds ratio; 95% CI, 95% Confidence Interval; P-value, probability value were calculated using Fisher’s exact test or Chi-square test; Pc-value, Corrected p-value were adjusted by Bonferroni’s correction (22 for HLA-B and 20 for HLA-C). In bold: Data analysis result was presented statistical significance (p-value < 0.05).

### Association Between Dapsone-Induced SJS-TEN and *HLA* Class I, II Alleles

The association between *HLA* class I and II alleles and dapsone-induced SJS-TEN is shown in **Table 5**. HLA-B*13:01 showed a significant association with dapsone-induced SJS-TEN in Thais. HLA-B*13:01 was observed in 80.00% (4/5) of patients with dapsone-induced SJS-TEN, but only in 10.00% (4/40) of tolerant controls (OR: 36.00; 95% CI: 3.19–405.89 and p-value = 2.1657 × 10^−3^) and 11.49% (54/470) of general Thai population (OR: 30.82; 95% CI: 3.38–280.78 and p-value = 9.199 × 10^−4^). HLA-B*15:02 allele was found in 40.00% (2/5) of patients with dapsone-induced SJS-TEN, 10% (4/40) of tolerant controls, and 15.11% (71/470) of the general Thai population. There was no significant association between the HLA-B*15:02 allele and the dapsone-induced SJS-TEN (**Table 5**). We also observed a significant association of HLA-DRB1*15:01, HLA-B*13:01–C*03:04, and HLA-B*13:01–DRB1*15:01 with dapsone-induced SJS-TEN when compared with tolerant controls and general Thai population (p < 0.05). After taking corrected p-values into account, only HLA-B*13:01 was significantly associated with dapsone-induced SJS-TEN in Thais. HLA-B*13:01 had a sensitivity of 50.00% and specificity of 97.30% as a predictor for dapsone-induced SJS-TEN in Thais. Also, the PPV and NPV of the HLA-B*13:01 were 20.80 and 99.28%, respectively (**Table 3**).

**Table 5:** Association of *HLA* class I and II alleles with dapsone-induced SJS-TEN.

Pharmacogenomics markers	Dapsone-induced SJS-TEN (n = 5)	Dapsone controls (n = 40)	Thai population (n = 470)	Dapsone-induced SJS-TEN cases *versus* controls			Dapsone-induced SJS-TEN cases *versus* Thais		
				Odds ratio (95% CI)	*P*-value	*P*c-value	Odds ratio (95% CI)	*P*-value	*P*c-value
***HLA class I***									
HLA-A*02:01	0	6 (15.00%)	51 (10.85%)	0.48 (0.02–9.83)	1.0000	NS	0.74 (0.04–13.59)	1.0000	NS
HLA-A*02:03	0	8 (20.00%)	99 (21.06%)	0.35 (0.02–6.93)	0.5675	NS	0.34 (0.02–6.19)	0.5887	NS
HLA-A*02:06	0	3 (7.50%)	21 (4.47%)	0.97 (0.04–21.52)	1.0000	NS	1.90 (0.10–35.49)	1.0000	NS
HLA-A*02:07	1 (20.00%)	9 (22.50%)	68 (14.47%)	0.86 (0.09–8.71)	1.0000	NS	1.48 (0.16–13.42)	0.5454	NS
HLA-A*11:01	4 (80.00%)	16 (40.00%)	211 (44.89%)	6.00 (0.61–58.71)	0.1553	NS	4.91 (0.55–44.26)	0.1809	NS
HLA-A*24:02	1 (20.00%)	5 (12.50%)	95 (20.21%)	1.75 (0.16–18.97)	0.5287	NS	0.99 (0.11–8.93)	1.0000	NS
HLA-A*24:07	1 (20.00%)	2 (5.00%)	39 (8.30%)	4.75 (0.35–64.74)	0.3037	NS	2.76 (0.30–25.33)	0.3571	NS
HLA-A*30:01	0	3 (7.50%)	20 (4.26%)	0.97 (0.04–21.52)	1.0000	NS	1.99 (0.11–37.37)	1.0000	NS
HLA-A*33:01	0	3 (7.50%)	3 (0.64%)	0.97 (0.04–21.52)	1.0000	NS	12.14 (0.56–264.26)	1.0000	NS
HLA-A*33:03	0	13 (32.50%)	99 (21.06%)	0.19 (0.01–3.60)	0.3007	NS	0.34 (0.02–6.19)	0.5887	NS
HLA-B*07:05	0	1 (2.50%)	24 (5.11%)	2.39 (0.09–66.39)	1.0000	NS	1.66 (0.09–30.83)	1.0000	NS
HLA-B*13:01	**4 (80.00%)**	**4 (10.00%)**	**54 (11.49%)**	**36.00 (3.19–405.89)**	**2.1657 × 10^—3^**	**4.7645 × 10^—2^**	**30.82 (3.38–280.78)**	**9.1996 × 10^—4^**	**2.0239 × 10^—2^**
HLA-B*13:02	0	3 (7.50%)	20 (4.26%)	0.97 (0.04–21.52)	1.0000	NS	1.99 (0.11–37.37)	1.0000	NS
HLA-B*15:02	2 (40.00%)	4 (10.00%)	71 (15.11%)	6.00 (0.76–47.36)	0.1248	NS	3.75 (0.62–22.82)	0.1709	NS
HLA-B*15:35	0	1 (2.50%)	3 (0.64%)	2.39 (0.09–66.39)	1.0000	NS	12.14 (0.56–264.26)	1.0000	NS
HLA-B*18:01	0	5 (12.50%)	36 (7.66%)	0.59 (0.03–12.16)	1.0000	NS	1.08 (0.06–19.96)	1.0000	NS
HLA-B*27:06	1 (20.00%)	2 (5.00%)	12 (2.55%)	4.75 (0.35–64.74)	0.3037	NS	9.54 (0.99–91.89)	0.1301	NS
HLA-B*38:02	0	4 (10.00%)	39 (8.30%)	0.74 (0.04–15.67)	1.0000	NS	0.99 (0.05–18.29)	1.0000	NS
HLA-B*40:01	1 (20.00%)	7 (17.50%)	58 (12.34%)	1.18 (0.11–12.21)	1.0000	NS	1.78 (0.19–16.16)	0.4863	NS
HLA-B*44:03	0	5 (12.50%)	42 (8.94%)	0.59 (0.03–12.16)	1.0000	NS	0.92 (0.05–16.86)	1.0000	NS
HLA-B*46:01	1 (20.00%)	10 (25.00%)	122 (25.96%)	0.75 (0.08–7.52)	1.0000	NS	0.71 (0.08–6.44)	1.0000	NS
HLA-B*51:01	0	3 (7.50%)	40 (8.51%)	0.97 (0.04–21.52)	1.0000	NS	0.97 (0.05–17.79)	1.0000	NS
HLA-B*58:01	0	5 (12.50%)	57 (12.13%)	0.59 (0.03–12.16)	1.0000	NS	0.65 (0.04–11.98)	1.0000	NS
HLA-C*01:02	1 (20.00%)	10 (25.00%)	143 (30.43%)	0.75 (0.08–7.52)	1.0000	NS	0.57 (0.06–5.16)	1.0000	NS
HLA-C*03:02	0	7 (17.50%)	69 (14.68%)	0.41 (0.02–8.17)	0.5771	NS	0.53 (0.03–9.61)	1.0000	NS
HLA-C*03:04	**3 (60.00%)**	**4 (10.00%)**	**66 (14.04%)**	**13.50 (1.71–106.56)**	**0.0212**	0.4249	**9.18 (1.51–55.99)**	**0.0237**	0.4734
HLA-C*03:09	**1 (20.00%)**	1 (2.50%)	**1 (0.21%)**	9.75 (0.51–187.53)	0.2121	NS	**117.25 (6.19–2220.85)**	**0.0210**	0.4193
HLA-C*04:01	0	2 (5.00%)	44 (9.36%)	1.40 (0.06–33.17)	1.0000	NS	0.87 (0.05–16.02)	1.0000	NS
HLA-C*06:02	0	4 (10.00%)	40 (8.51%)	0.74 (0.04–15.67)	1.0000	NS	0.97 (0.05–17.79)	1.0000	NS
HLA-C*07:01	0	7 (17.50%)	58 (12.34%)	0.41 (0.02–8.17)	0.5771	NS	0.64 (0.04–11.74)	1.0000	NS
HLA-C*07:02	0	10 (25.00%)	101 (21.49%)	0.26 (0.01–5.19)	0.5714	NS	0.33 (0.02–6.04)	0.5893	NS
HLA-C*07:04	1 (20.00%)	6 (15.00%)	46 (9.79%)	1.42 (0.13–14.96)	1.0000	NS	2.30 (0.25–21.06)	0.4074	NS
HLA-C*08:01	2 (40.00%)	7 (17.50%)	90 (19.15%)	3.14 (0.44–22.45)	0.2575	NS	2.82 (0.46–17.09)	0.2494	NS
***HLA class II***									
HLA-DRB1*03:01	0	6 (15.00%)	43 (9.15%)	0.48 (0.02–9.83)	1.0000	NS	0.89 (0.05–16.43)	1.0000	NS
HLA-DRB1*04:05	1 (20.00%)	1 (2.50%)	45 (9.57%)	9.75 (0.51–187.53)	0.2121	NS	2.36 (0.26–21.58)	0.4004	NS
HLA-DRB1*07:01	0	9 (22.50%)	83 (17.66%)	0.30 (0.02–5.96)	0.5661	NS	0.42 (0.02–7.70)	0.5924	NS
HLA-DRB1*08:03	0	1 (2.50%)	14 (2.98%)	2.39 (0.09–66.39)	1.0000	NS	2.86 (0.15–54.24)	1.0000	NS
HLA-DRB1*09:01	0	2 (5.00%)	88 (18.72%)	1.40 (0.06–33.17)	1.0000	NS	0.39 (0.02–7.17)	0.5896	NS
HLA-DRB1*11:01	1 (20.00%)	1 (2.50%)	15 (3.19%)	9.75 (0.51–187.53)	0.2121	NS	7.58 (0.79–72.01)	0.1581	NS
HLA-DRB1*12:02	1 (20.00%)	8 (20.00%)	134 (28.51%)	1.00 (0.09–10.22)	1.0000	NS	0.63 (0.07–5.66)	1.0000	NS
HLA-DRB1*14:01	1 (20.00%)	8 (20.00%)	51 (10.85%)	1.00 (0.09–10.22)	1.0000	NS	2.05 (0.23–18.73)	0.4414	NS
HLA-DRB1*15:01	**3 (60.00%)**	**5 (12.50%)**	**72 (15.32%)**	**10.50 (1.39–79.13)**	**0.0327**	0.5885	**8.29 (1.36–50.50)**	**0.0299**	0.5375
HLA-DRB1*15:02	0	14 (35.00%)	124 (26.38%)	0.17 (0.01–3.22)	0.3046	NS	0.25 (0.01–4.61)	0.3331	NS
HLA-DRB1*16:02	1 (20.00%)	9 (22.50%)	52 (11.06%)	0.86 (0.09–8.71)	1.0000	NS	2.01 (0.22–18.32)	0.4480	NS
HLA-DQA1*01:01	1 (20.00%)	22 (55.00%)	196 (41.70%)	0.21 (0.02–1.99)	0.1868	NS	0.35 (0.04–3.15)	0.4085	NS
HLA-DQA1*01:02	3 (60.00%)	18 (45.00%)	183 (38.94%)	1.83 (0.28–12.19)	0.6521	NS	2.35 (0.39–14.21)	0.3845	NS
HLA-DQA1*01:03	0	3 (7.50%)	34 (7.23%)	0.97 (0.04–21.52)	1.0000	NS	1.15 (0.06–21.24)	1.0000	NS
HLA-DQA1*02:01	0	6 (15.00%)	81 (17.23%)	0.48 (0.02–9.83)	1.0000	NS	0.44 (0.02–7.94)	0.5940	NS
HLA-DQA1*03:01	0	1 (2.50%)	40 (8.51%)	2.39 (0.09–66.39)	1.0000	NS	0.97 (0.05–17.79)	1.0000	NS
HLA-DQA1*03:02	1 (20.00%)	4 (10.00%)	125 (26.60%)	2.25 (0.20–25.37)	0.4614	NS	0.69 (0.08–6.23)	1.0000	NS
HLA-DQA1*05:01	0	7 (17.50%)	49 (10.43%)	0.41 (0.02–8.17)	0.5771	NS	0.77 (0.04–14.21)	1.0000	NS
HLA-DQA1*05:05	**2 (40.00%)**	3 (7.50%)	**31 (6.60%)**	8.22 (0.97–69.98)	0.0874	NS	**9.44 (1.52–58.61)**	**0.0410**	0.3694
HLA-DQA1*06:01	1 (20.00%)	7 (17.50%)	107 (22.77%)	1.18 (0.11–12.21)	1.0000	NS	0.85 (0.09–7.67)	1.0000	NS
HLA-DQB1*02:01	0	6 (15.00%)	47 (10.00%)	0.48 (0.02–9.83)	1.0000	NS	0.81 (0.04–14.89)	1.0000	NS
HLA-DQB1*02:02	0	8 (20.00%)	68 (14.47%)	0.35 (0.02–6.93)	0.5675	NS	0.53 (0.03–9.77)	1.0000	NS
HLA-DQB1*03:01	2 (40.00%)	11 (27.50%)	151 (32.13%)	1.76 (0.26–11.98)	0.6174	NS	1.41 (0.23–8.52)	0.6591	NS
HLA-DQB1*03:02	0	2 (5.00%)	37 (7.87%)	1.40 (0.06–33.17)	1.0000	NS	1.05 (0.06–19.37)	1.0000	NS
HLA-DQB1*03:03	0	3 (7.50%)	101 (21.49%)	0.97 (0.04–21.52)	1.0000	NS	0.33 (0.02–6.04)	0.5893	NS
HLA-DQB1*04:01	1 (20.00%)	1 (2.50%)	35 (7.45%)	9.75 (0.51–187.53)	0.2121	NS	3.11 (0.34–28.56)	0.3269	NS
HLA-DQB1*05:01	0	10 (25.00%)	121 (25.74%)	0.26 (0.01–5.19)	0.5714	NS	0.26 (0.01–4.76)	0.3357	NS
HLA-DQB1*05:02	2 (40.00%)	19 (47.50%)	182 (38.72%)	0.74 (0.11–4.89)	1.0000	NS	1.06 (0.18–6.37)	1.0000	NS
HLA-DQB1*05:03	0	6 (15.00%)	37 (7.87%)	0.48 (0.02–9.83)	1.0000	NS	1.05 (0.06–19.37)	1.0000	NS
HLA-DQB1*06:01	2 (40.00%)	5 (12.50%)	63 (13.40%)	4.67 (0.62–35.17)	0.1662	NS	4.31 (0.71–26.28)	0.1402	NS
HLA-DQB1*06:02	0	1 (2.50%)	14 (2.98%)	2.39 (0.09–66.39)	1.0000	NS	2.86 (0.15–54.24)	1.0000	NS
**Haplotype**									
HLA-B*13:01/ C*03:04	**3 (60.00%)**	**2 (5.00%)**	**31 (6.60%)**	**28.50 (2.89–280.14)**	**0.0065**	0.2750	**21.24 (3.42–131.88)**	**0.0030**	0.1280
HLA-B*13:01/ DRB1*15:01	**2 (40.00%)**	2 (5.00%)	**12 (2.55%)**	12.67 (1.29–124.51)	0.0551	NS	**25.44 (3.89–166.54)**	**0.0077**	0.3072
HLA-B*13:01/ DQB1*06:01	1 (20.00%)	1 (2.50%)	9 (1.91%)	9.75 (0.51–187.53)	0.2121	NS	12.81 (1.29–126.26)	0.1013	NS
HLA-B*13:01/ DRB1*15:01/ DQB1*06:01	1 (20.00%)	1 (2.50%)	7 (1.49%)	9.75 (0.51–187.53)	0.2121	NS	16.54 (1.63–167.41)	0.0818	NS
HLA-DRB1*15:01/ DQB1*06:01	**2 (40.00%)**	3 (7.50%)	**33 (7.02%)**	8.22 (0.97–69.98)	0.0874	NS	**8.83 (1.43–54.69)**	**0.0458**	NS

Significant different P-value <0.05; HLA-A, human leucocyte antigen-A; HLA-B, human leucocyte antigen-B; HLA-C, human leucocyte antigen-C; HLA-DRB1, human leukocyte antigen-DRB1; HLA-DQA1, human leucocyte antigen-DQA1; HLA-DQB1, human leucocyte antigen-DQB1; SJS, Stevens-Johnson syndrome; TEN, toxic epidermal necrolysis; OR, odds ratio; 95% CI, 95% Confidence Interval; P-value, probability value were calculated using Fisher’s exact test or Chi-square test; Pc-value, Corrected p-value were adjusted by Bonferroni’s correction (16 for HLA-A, 22 for HLA-B, 20 for HLA-C, 18 for HLA-DRB1, 9 for HLA-DQA1, and 11 for HLA-DQB1); NS, Not significant. In bold: Data analysis result was presented statistical significance (p-value < 0.05).

When we compared the frequency of HLA-B*13:01 allele of seven Asian patients with dapsone-induced SJS-TEN and dapsone-tolerant control Thais and the general Thai population, HLA-B*13:01 allele was strongly associated with dapsone-induced SJS-TEN among Asians compared to the dapsone-tolerant control Thais (OR: 54.00; 95% CI: 5.12–569.39 and *P*c-value = 2.7599 × 10^−3^) and general Thai population (OR: 46.22; 95% CI: 5.46–391.26 and *P*c-value = 4.3858 × 10^−4^) respectively (**Table 4**). For the Taiwanese study, the results showed a significant association between HLA-B*13:01 allele and dapsone-induced SJS/TEN when compared with Thai tolerant control groups with an OR of 40.56 (95% CI = 1.67–985.44; p = 0.0174).

### Association Between Dapsone-Induced DRESS and *HLA* Class I, II Alleles

The association of *HLA* class I and II alleles with dapsone-induced DRESS were shown in **Table 6**. We found that 81.82% (9/11) of dapsone-induced DRESS cases carried HLA-B*13:01, while 10.00% (4/40) of dapsone-tolerant controls and 11.49% (54/470) of the general Thai population carried HLA-B*13:01 allele. The HLA-B*13:01 allele was significantly associated with dapsone-induced DRESS when compared with dapsone-tolerant controls (OR: 40.50; 95% CI: 6.38–257.03 and *p*-value = 1.0784 × 10^−5^) and general Thai population (OR: 34.67; 95% CI: 7.29–164.67 and *p*-value = 2.9734 × 10^−7^). These results were confirmed by corrected *p*-value of *HLA-B* alleles (2.3725 × 10^−4^ and 6.5415 × 10^−6^, respectively) as presented in the **Table 6**. The sensitivity, specificity, PPV, and NPV of HLA-B*13:01 allele and dapsone-induced DRESS patients was 69.23, 94.74, 15.74, and 99.54%, respectively (**Table 3**).

**Table 6:** Association of *HLA* class I and II alleles with dapsone-induced DRESS.

Pharmacogenomics markers	Dapsone-induced DRESS (n = 11)	Dapsone controls (n = 40)	Thai population (n = 470)	Dapsone-induced DRESS cases *versus* controls			Dapsone-induced DRESS cases *versus* Thais		
				Odds ratio (95% CI)	*P*-value	*Pc*- value	Odds ratio (95% CI)	*P*-value	*Pc*-value
***HLA class I***									
HLA-A*02:01	0	6 (15.00%)	51 (10.85%)	0.23 (0.01–4.42)	0.3190	NS	0.35 (0.02–6.09)	0.6160	NS
HLA-A*02:03	1 (9.09%)	8 (20.00%)	99 (21.06%)	0.40 (0.04–3.59)	0.6630	NS	0.38 (0.05–2.96)	0.4729	NS
HLA-A*02:06	0	3 (7.50%)	21 (4.47%)	0.47 (0.02–9.69)	1.0000	NS	0.91 (0.05–15.94)	1.0000	NS
HLA-A*02:07	1 (9.09%)	9 (22.50%)	68 (14.47%)	0.34 (0.04–3.06)	0.4282	NS	0.59 (0.07–4.69)	1.0000	NS
HLA-A*11:01	6 (54.55%)	16 (40.00%)	211 (44.89%)	1.80 (0.47–6.91)	0.4976	NS	1.47 (0.44–4.89)	0.5546	NS
HLA-A*24:02	3 (27.27%)	5 (12.50%)	95 (20.21%)	2.63 (0.52–13.32)	0.3464	NS	1.48 (0.39–5.69)	0.4743	NS
HLA-A*24:07	3 (27.27%)	2 (5.00%)	39 (8.30%)	7.13 (1.01–49.82)	0.0606	0.9697	4.14 (1.06–16.26)	0.0623	0.9974
HLA-A*30:01	1 (9.09%)	3 (7.50%)	20 (4.26%)	1.23 (0.12–13.17)	1.0000	NS	2.25 (0.27–18.45)	0.3913	NS
HLA-A*33:01	0	3 (7.50%)	3 (0.64%)	0.47 (0.02–9.69)	1.0000	NS	5.81 (0.28–119.05)	1.0000	NS
HLA-A*33:03	1 (9.09%)	13 (32.50%)	99 (21.06%)	0.21 (0.02–1.80)	0.2508	NS	0.38 (0.05–2.96)	0.4730	NS
HLA-B*07:05	1 (9.09%)	1 (2.50%)	24 (5.11%)	3.90 (0.22–67.93)	0.3882	NS	1.86 (0.23–15.12)	0.4476	NS
HLA-B*13:01	**9 (81.82%)**	**4 (10.00%)**	**54 (11.49%)**	**40.50 (6.38–257.03)**	**1.0784 × 10^−5^**	**2.3725 × 10^−4^**	**34.67 (7.29–164.67)**	**2.9734 × 10^−7^**	**6.5415 × 10^−6^**
HLA-B*13:02	1 (9.09%)	3 (7.50%)	20 (4.26%)	1.23 (0.12–13.17)	1.0000	NS	2.25 (0.27–18.45)	0.3913	NS
HLA-B*15:02	3 (27.27%)	4 (10.00%)	71 (15.11%)	3.38 (0.63–18.14)	0.1617	NS	2.11 (0.55–8.14)	0.3873	NS
HLA-B*15:35	1 (9.09%)	1 (2.50%)	3 (0.64%)	3.90 (0.22–67.93)	0.3882	NS	15.57 (1.49–162.94)	0.0887	NS
HLA-B*18:01	1 (9.09%)	5 (12.50%)	36 (7.66%)	0.70 (0.07–6.70)	1.0000	NS	1.21 (0.15–9.68)	0.5894	NS
HLA-B*27:06	0	2 (5.00%)	12 (2.55%)	0.67 (0.03–14.97)	1.0000	NS	1.59 (0.09–28.60)	1.0000	NS
HLA-B*38:02	0	4 (10.00%)	39 (8.30%)	0.35 (0.02–7.06)	0.5651	NS	0.48 (0.03–8.21)	1.0000	NS
HLA-B*40:01	0	7 (17.50%)	58 (12.34%)	0.19 (0.01–3.67)	0.3227	NS	0.31 (0.02–5.27)	0.3754	NS
HLA-B*44:03	0	5 (12.50%)	42 (8.94%)	0.28 (0.01–5.47)	0.5720	NS	0.44 (0.03–7.57)	0.6102	NS
HLA-B*46:01	1 (9.09%)	10 (25.00%)	122 (25.96%)	0.30 (0.03–2.65)	0.4178	NS	0.29 (0.04–2.25)	0.3038	NS
HLA-B*51:01	0	3 (7.50%)	40 (8.51%)	0.47 (0.02–9.69)	1.0000	NS	0.46 (0.03–7.99)	0.6113	NS
HLA-B*58:01	1 (9.09%)	5 (12.50%)	57 (12.13%)	0.70 (0.07–6.70)	1.0000	NS	0.73 (0.09–5.77)	1.0000	NS
HLA-C*01:02	1 (9.09%)	10 (25.00%)	143 (30.43%)	0.30 (0.03–2.65)	0.4178	NS	0.23 (0.03–1.80)	0.1862	NS
HLA-C*03:02	1 (9.09%)	7 (17.50%)	69 (14.68%)	0.47 (0.05–4.30)	0.6685	NS	0.58 (0.07–4.61)	1.0000	NS
HLA-C*03:04	**5 (45.45%)**	**4 (10.00%)**	**66 (14.04%)**	**7.50 (1.56–36.17)**	**0.0155**	0.3093	**5.10 (1.51–17.19)**	**0.0138**	0.2752
HLA-C*03:09	**1 (9.09%)**	1 (2.50%)	**1 (0.21%)**	3.90 (0.22–67.93)	0.3882	NS	**46.90 (2.74–804.09)**	**0.0453**	0.9052
HLA-C*04:01	1 (9.09%)	2 (5.00%)	44 (9.36%)	1.90 (0.16–23.14)	0.5256	NS	0.97 (0.12–7.74)	1.0000	NS
HLA-C*06:02	1 (9.09%)	4 (10.00%)	40 (8.51%)	0.90 (0.09–8.98)	1.0000	NS	1.08 (0.13–8.61)	1.0000	NS
HLA-C*07:01	0	7 (17.50%)	58 (12.34%)	0.19 (0.01–3.67)	0.3227	NS	0.31 (0.02–5.27)	0.3754	NS
HLA-C*07:02	1 (9.09%)	10 (25.00%)	101 (21.49%)	0.30 (0.03–2.65)	0.4178	NS	0.37 (0.05–2.89)	0.4712	NS
HLA-C*07:04	1 (9.09%)	6 (15.00%)	46 (9.79%)	0.57 (0.06–5.28)	1.0000	NS	0.92 (0.12–7.36)	1.0000	NS
HLA-C*08:01	4 (36.36%)	7 (17.50%)	90 (19.15%)	2.69 (0.62–11.77)	0.2220	NS	2.41 (0.69–8.42)	0.2377	NS
***HLA class II***									
HLA-DRB1*03:01	1 (9.09%)	6 (15.00%)	43 (9.15%)	0.57 (0.06–5.28)	1.0000	NS	0.99 (0.12–7.94)	1.0000	NS
HLA-DRB1*04:05	1 (9.09%)	1 (2.50%)	45 (9.57%)	3.90 (0.22–67.93)	0.3882	NS	0.94 (0.12–7.55)	1.0000	NS
HLA-DRB1*07:01	1 (9.09%)	9 (22.50%)	83 (17.66%)	0.34 (0.04–3.06)	0.4282	NS	0.47 (0.06–3.69)	0.6983	NS
HLA-DRB1*08:03	**2 (18.18%)**	1 (2.50%)	**14 (2.98%)**	8.67 (0.71–106.38)	0.1136	NS	**7.24 (1.43–36.64)**	**0.0479**	0.8627
HLA-DRB1*09:01	2 (18.18%)	2 (5.00%)	88 (18.72%)	4.22 (0.52–34.15)	0.1994	NS	0.97 (0.21–4.54)	1.0000	NS
HLA-DRB1*11:01	1 (9.09%)	1 (2.50%)	15 (3.19%)	3.90 (0.22–67.93)	0.3882	NS	3.03 (0.36–25.25)	0.3135	NS
HLA-DRB1*12:02	1 (9.09%)	8 (20.00%)	134 (28.51%)	0.40 (0.04–3.59)	0.6630	NS	0.25 (0.03–1.98)	0.3055	NS
HLA-DRB1*14:01	1 (9.09%)	8 (20.00%)	51 (10.85%)	0.40 (0.04–3.59)	0.6630	NS	0.82 (0.10–6.55)	1.0000	NS
HLA-DRB1*15:01	4 (36.36%)	5 (12.50%)	72 (15.32%)	4.00 (0.85–18.75)	0.0868	NS	3.16 (0.90–11.07)	0.0791	NS
HLA-DRB1*15:02	2 (18.18%)	14 (35.00%)	124 (26.38%)	0.41 (0.08–2.18)	0.4663	NS	0.62 (0.13–2.91)	0.7358	NS
HLA-DRB1*16:02	1 (9.09%)	9 (22.50%)	52 (11.06%)	0.34 (0.04–3.06)	0.4282	NS	0.80 (0.10–6.41)	1.0000	NS
HLA-DQA1*01:01	4 (36.36%)	22 (55.00%)	196 (41.70%)	0.47 (0.12–1.85)	0.2735	NS	0.79 (0.23–2.77)	1.0000	NS
HLA-DQA1*01:02	5 (45.45%)	18 (45.00%)	183 (38.94%)	1.02 (0.27–3.89)	1.0000	NS	1.31 (0.39–4.34)	0.7577	NS
HLA-DQA1*01:03	2 (18.18%)	3 (7.50%)	34 (7.23%)	2.74 (0.39–18.92)	0.2919	NS	2.85 (0.59–13.72)	0.1957	NS
HLA-DQA1*02:01	0	6 (15.00%)	81 (17.23%)	0.23 (0.01–4.42)	0.3190	NS	0.21 (0.01–3.56)	0.2246	NS
HLA-DQA1*03:01	1 (9.09%)	1 (2.50%)	40 (8.51%)	3.90 (0.22–67.93)	0.3882	NS	1.08 (0.13–8.61)	1.0000	NS
HLA-DQA1*03:02	2 (18.18%)	4 (10.00%)	125 (26.60%)	2.00 (0.32–12.69)	0.5981	NS	0.61 (0.13–2.88)	0.7355	NS
HLA-DQA1*05:01	1 (9.09%)	7 (17.50%)	49 (10.43%)	0.47 (0.05–4.30)	0.6685	NS	0.86 (0.11–6.86)	1.0000	NS
HLA-DQA1*05:05	2 (18.18%)	3 (7.50%)	31 (6.60%)	2.74 (0.39–18.92)	0.2919	NS	3.15 (0.65–15.20)	0.1703	NS
HLA-DQA1*06:01	1 (9.09%)	7 (17.50%)	107 (22.77%)	0.47 (0.05–4.30)	0.6685	NS	0.34 (0.04–2.68)	0.4693	NS
HLA-DQB1*02:01	1 (9.09%)	6 (15.00%)	47 (10.00%)	0.57 (0.06–5.28)	1.0000	NS	0.90 (0.11–7.19)	1.0000	NS
HLA-DQB1*02:02	1 (9.09%)	8 (20.00%)	68 (14.47%)	0.40 (0.04–3.59)	0.6630	NS	0.59 (0.07–4.69)	1.0000	NS
HLA-DQB1*03:01	3 (27.27%)	11 (27.50%)	151 (32.13%)	0.99 (0.22–4.42)	1.0000	NS	0.79 (0.21–3.03)	1.0000	NS
HLA-DQB1*03:02	2 (18.18%)	2 (5.00%)	37 (7.87%)	4.22 (0.52–34.15)	0.1994	NS	2.60 (0.54–12.48)	0.2218	NS
HLA-DQB1*03:03	2 (18.18%)	3 (7.50%)	101 (21.49%)	2.74 (0.39–18.92)	0.2919	NS	0.81 (0.17–3.82)	1.0000	NS
HLA-DQB1*04:01	0	1 (2.50%)	35 (7.45%)	1.15 (0.04–30.05)	1.0000	NS	0.53 (0.03–9.24)	1.0000	NS
HLA-DQB1*05:01	2 (18.18%)	10 (25.00%)	121 (25.74%)	0.67 (0.12–3.62)	1.0000	NS	0.64 (0.14–3.01)	0.7371	NS
HLA-DQB1*05:02	4 (36.36%)	19 (47.50%)	182 (38.72%)	0.63 (0.16–2.50)	0.7338	NS	0.90 (0.26–3.13)	1.0000	NS
HLA-DQB1*05:03	1 (9.09%)	6 (15.00%)	37 (7.87%)	0.57 (0.06–5.28)	1.0000	NS	1.17 (0.15–9.39)	0.5996	NS
HLA-DQB1*06:01	**5 (45.45%)**	**5 (12.50%)**	**63 (13.40%)**	**5.83 (1.29–26.46)**	**0.0274**	0.3010	**5.38 (1.59–18.17)**	**0.0114**	0.1255
HLA-DQB1*06:02	1 (9.09%)	1 (2.50%)	14 (2.98%)	3.90 (0.22–67.93)	0.3882	NS	3.26 (0.39–27.23)	0.2969	NS
**Haplotype**									
HLA-B*13:01/ C*03:04	**5 (45.45%)**	**2 (5.00%)**	**31 (6.60%)**	**15.83 (2.48–100.91)**	**0.0033**	0.1386	**11.80 (3.41–40.84)**	**5.9299 × 10^−4^**	**0.0249**
HLA-B*13:01/ DRB1*15:01	**3 (27.27%)**	2 (5.00%)	**12 (2.55%)**	7.13 (1.02–49.82)	0.0606	NS	**14.31 (3.37–60.74)**	**0.0035**	0.1400
HLA-B*13:01/ DQB1*06:01	**4 (36.36%)**	**1 (2.50%)**	**9 (1.91%)**	**22.29 (2.16–230.05)**	**0.0058**	0.1914	**29.27 (7.26–118.03)**	**9.6269 × 10^−5^**	**0.0032**
HLA-B*13:01/ DRB1*15:01/ DQB1*06:01	**2 (18.18%)**	1 (2.50%)	**7 (1.49%)**	8.67 (0.71–106.38)	0.1136	NS	**14.69 (2.67–80.81)**	**0.0157**	0.8007
HLA-DRB1*15:01/ DQB1*06:01	**3 (27.27%)**	3 (7.50%)	**33 (7.02%)**	4.63 (0.79–27.25)	0.1059	NS	**4.97 (1.26–19.61)**	**0.0419**	NS

Significant different p-value <0.05; HLA-A, human leucocyte antigen-A; HLA-B, human leucocyte antigen-B; HLA-C, human leucocyte antigen-C; HLA-DRB1, human leukocyte antigen-DRB1; HLA-DQA1, human leucocyte antigen-DQA1; HLA-DQB1, human leucocyte antigen-DQB1; DRESS, drug reaction with eosinophilia and systemic symptoms; OR, odds ratio; 95% CI, 95% Confidence Interval; P-value, probability value were calculated using Fisher’s exact test or Chi-square test; Pc-value, Corrected p-value were adjusted by Bonferroni’s correction (16 for HLA-A, 22 for HLA-B, 20 for HLA-C, 18 for HLA-DRB1, 9 for HLA-DQA1, and 11 for HLA-DQB1); NS, Not significant. In bold: Data analysis result was presented statistical significance (p-value < 0.05).

On comparing, 11 dapsone-induced DRESS cases with 40 tolerant controls and 470 general Thai population, the frequencies of HLA-C*03:04, HLA-DQB1*06:01, HLA-B*13:01–C*03:04, and HLA-B*13:01–DQB1*06:01 were significantly associated with dapsone-induced DRESS (*p*-value < 0.05). However, HLA-B*15:02 allele was not statistically significant association with dapsone-induced DRESS when compared with tolerant controls and Thai population by *p*-value of 0.1617 and 0.3873, respectively. When the frequencies of *HLA* alleles in Asian and Taiwanese group were compared with those in Thai dapsone-tolerant controls and the general Thai population, only the HLA-B*13:01 allele was associated with dapsone-induced DRESS (**Table 4**). Whereas the HLA-C*03:04 allele was not statistically significant in this subgroup.

### Association Between Dapsone-Induced SCARs and Cytochrome P450 (*CYP2C9*, *CYP2C19*, and *CYP3A4* Variants)

In this study, none of the dapsone-induced SCARs and subgroups carried CYP2C9*2 variant along with tolerant controls. CYP2C9*3 variant (intermediate metabolizer, IM) was found in 6.25% (1/16) of the patients with dapsone-induced SCARs and 12.50% (5/40) of the dapsone-tolerant controls. Dapsone-induced SCARs and subgroups were not significantly associated with CYP2C9*3 variant (*p*-value = 0.6622 and 1.0000) as shown in the **Table 7**. There were no significant association between *CYP2C19* variant and dapsone-induced SCARs and subgroup. CYP3A4*1B variant was absent in this study population. We found one individual of dapsone-induced SCARs carrying CYP3A4*1/*18. There were not significantly associated between *CY3A4* variant and dapsone-induced SCARs and subgroups in Thais.

**Table 7:** Association of Cytochrome P450 (*CYP2C9*, *CYP2C19*, and *CYP3A4*) with dapsone-induced SCARs.

Phenotype	Cytochrome P450	Dapsone cases n (%)	Dapsone controls n (%)	OR (95% CI)	*P*-value	*Pc*-value
SCARs (n = 16)	*CYP2C9*					
	*1/*1	15 (93.75)	35 (87.50)			
	*1/*3	1 (6.25)	5 (12.50)	0.47 (0.05–4.34)	0.6622	NS
	*CYP2C19*					
	*1/*1	6 (37.50)	14 (35.00)			
	*1/*2	6 (37.50)	17 (42.50)	0.81 (0.25–2.67)	0.7312	NS
	*1/*3	2 (12.50)	3 (7.50)	1.76 (0.27–11.69)	0.6172	NS
	*2/*2	1 (6.25)	4 (10.00)	0.60 (0.06–5.82)	1.0000	NS
	*2/*3	1 (6.25)	0	7.84 (0.30–202.93)	0.2857	0.8571
	*1/*17	0	2 (5.00)	0.47 (0.02–10.26)	1.0000	NS
	*CYP3A4*					
	*1/*1	15 (93.75)	40 (100)			
	*1/*18	1 (6.25)	0	7.84 (0.30–202.93)	0.2857	0.5714
SJS-TEN (n = 5)	*CYP2C9*					
	*1/*1	5 (100)	35 (87.50)			
	*1/*3	0	5 (12.50)	0.59 (0.03–12.16)	1.0000	NS
	*CYP2C19*					
	*1/*1	4 (80.00)	14 (35.00)			
	*1/*2	1 (20.00)	17 (42.50)	0.34 (0.04–3.30)	0.6337	NS
	*1/*17	0	2 (5.00)	1.40 (0.06–33.17)	1.0000	NS
	*CYP3A4*					
	*1/*1	5 (100)	40 (100)			
DRESS (n = 11)	*CYP2C9*					
	*1/*1	10 (90.91)	35 (87.50)			
	*1/*3	1 (9.09)	5 (12.50)	0.70 (0.07–6.70)	1.0000	NS
	*CYP2C19*					
	*1/*1	2 (18.18)	14 (35.00)			
DRESS (n = 11)	*CYP2C19*					
	*1/*2	5 (45.45)	17 (42.50)	1.13 (0.29–4.32)	1.0000	NS
	*1/*3	2 (18.18)	3 (7.50)	2.74 (0.39–18.92)	0.2919	0.8758
	*2/*2	1 (9.09)	4 (10.00)	0.90 (0.09–8.98)	1.0000	NS
	*2/*3	1 (9.09)	0	11.57 (0.44–305.02)	0.2157	0.6471
	*1/*17	0	2 (5.00)	0.67 (0.03–14.97)	1.0000	NS
	*CYP3A4*					
	*1/*1	10 (90.91)	40 (100)			
	*1/*18	1 (9.09)	0	11.57 (0.44–305.02)	0.2157	0.4314

Significant different p-value <0.05; SCARs, severe cutaneous adverse reactions; SJS, Stevens-Johnson syndrome; TEN, toxic epidermal necrolysis; DRESS, drug reaction with eosinophilia and systemic symptoms; OR, odds ratio; 95% CI, 95% Confidence Interval; P-value, probability value were calculated using Fisher’s exact test or Chi-square test; Pc-value, Corrected p-value were adjusted by Bonferroni’s correction (two for CYP2C9, three for CYP2C19, and two for CYP3A4); NS, Not significant. Such as CYP2C9*1 was presented wild type and *2 was presented variants and associated with SNP (single nucleotide polymorphism).

### Structure Activity Relationship of Dapsone and DDS-NHOH With HLA-B*13:01 by *In Silico* Model

**Figure 1:**
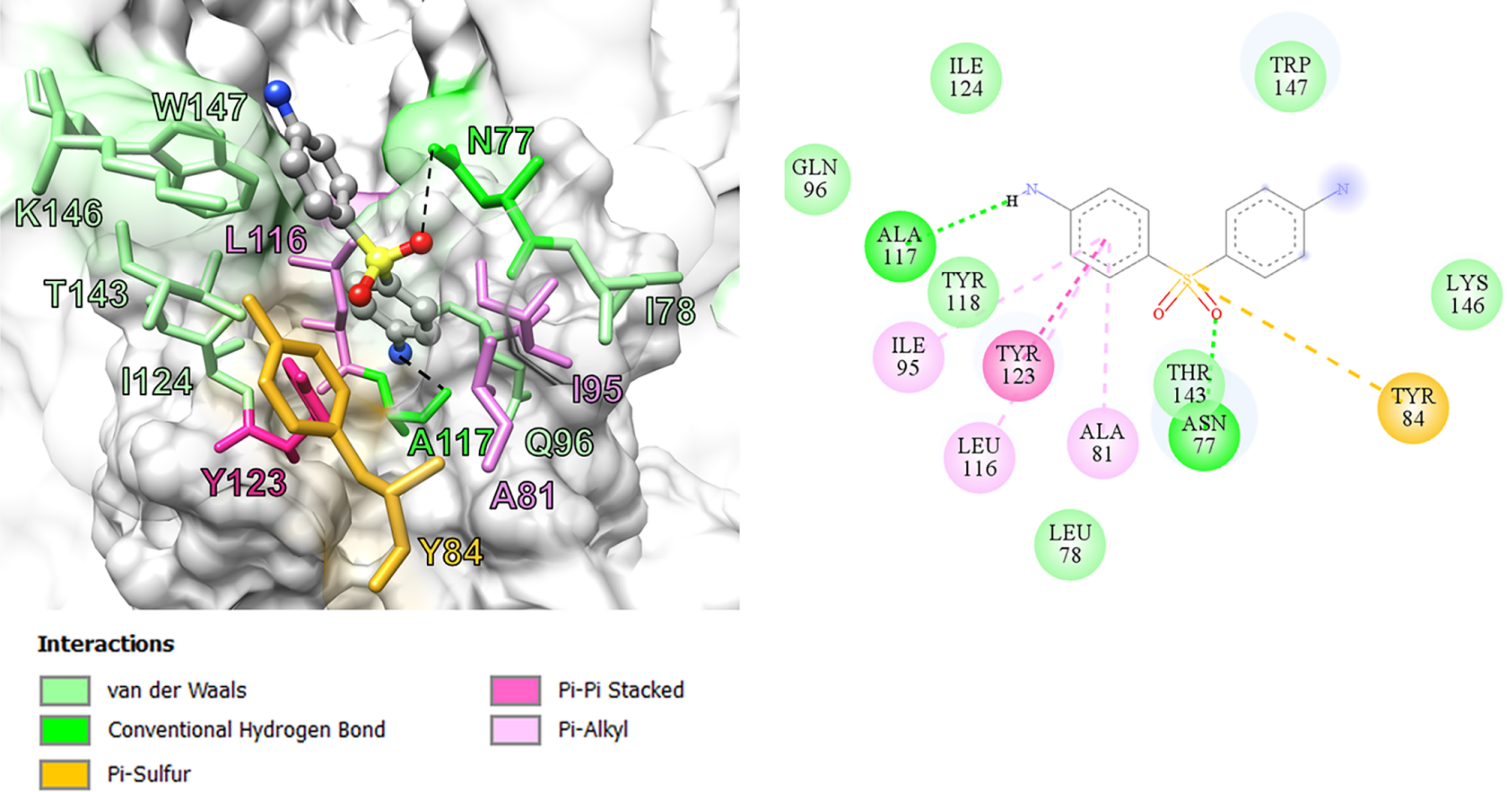
Binding model and interaction diagram between Dapsone and HLA-B*13:01.

**Figure 2:**
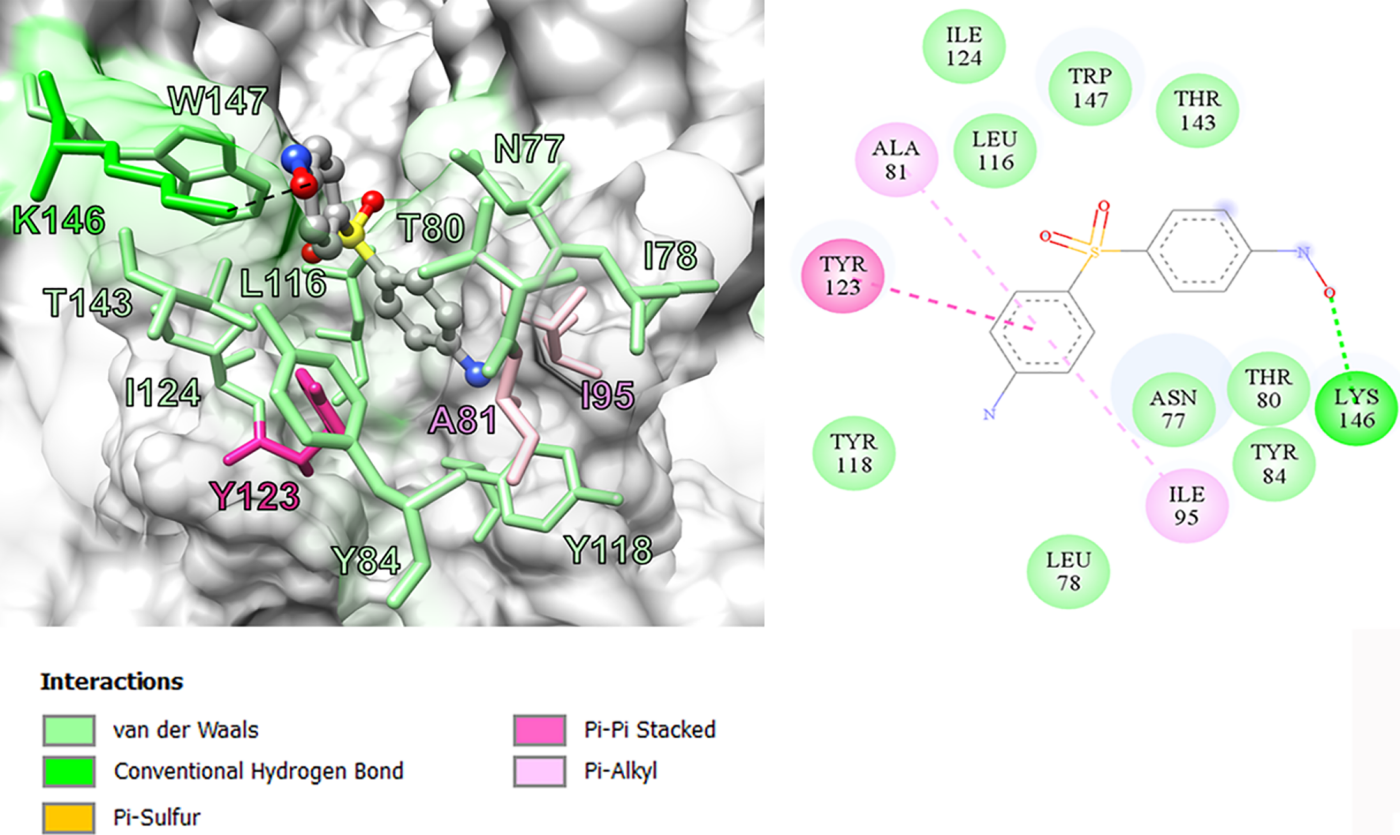
Binding model and interaction diagram between DDS-NHOH and HLA-B*13:01.

In this study, we performed computational analyses of dapsone and DDS-NHOH interacting with HLA-B*13:01 and -13:02 allele using the molecular docking approach by CDOCKER in Discovery Studio 2.5 program package. The homology models of HLA-B*13:01 and -13:02 were constructed by using HLA-B*5201 (PDB ID: 3W39) as the template structure. The 3D structure of either HLA-B*13:01 or -13:02 was deposited as a heterodimer containing α-domain and β-domain. In comparison between these two proteins, there are three different amino acids in the antigen-binding site of α-domain (I94, I95, and R97 in HLA-B*13:01, and T94, W95, and T97 in HLA-B*13:02). As a result, HLA-B*13:01 had an extra deep sub-pocket around the F-pocket at antigen-binding site, in which both drugs favorably occupied (**Figures 1** and **2**). The docking results in **Table 8** showed that although dapsone likely interacted with both proteins *via* an insertion of its –NH_2_ group into the F-pocket (90.4 and 100.0% for HLA-B*13:01 and -13:02), it preferred to bind with HLA-B*13:01 (−28.53 kcal/mol) more than HLA-B*13:02 (−25.19 kcal/mol). The functional substitution on one of –NH_2_ groups to the –NHOH group in DDS-NHOH could lead to a more stable complex with HLA-B*13:01 (−30.24 kcal/mol for the conformation with –NH_2_ insertion, 54.0%), however the complex with the –NHOH insertion was also possible (30.0%) but it was less stable (−27.45 kcal/mol). This is in contrast for DDS-NHOH/HLA-B*13:02 in which only the conformation with –NHOH insertion was detected (−26.42 kcal/mol) in the F-pocket at antigen-binding site.

**Table 8:** Binding free energies of dapsone and DDS-NHOH with HLA-B*13:01 and HLA-B*13:02.

Drug	HLA-B*13:01			HLA-B*13:02		
	CDOCKER-INTERACTION Energy	Structure binding	Number (%)	CDOCKER-INTERACTION Energy	Structure binding	Number (%)
**Dapsone**	-28.53	NH2	90.4%	-25.19	NH2	100.0%
	-26.86	NH2-NH2	6.4%			
	-16.82	SO2	3.2%			
**DDS-NHOH**	-30.24	NH2	54%	-26.42	NHOH	100.0%
	-27.45	NHOH	30%			
	-23.40	NH2-NHOH	16%			

## Discussion

The immunopathogenesis of SCARs are associated with expression of specific HLA allele, T-lymphocyte, structure of drug and peptide molecules (34, 35). In this study, we presented the highly specific association of HLA-B*13:01 allele and dapsone-induced SCARs (OR = 39.00, *p*-value = 5.3447 × 10^−7^), dapsone-induced SJS-TEN (OR = 36.00, *p*-value = 2.1657 × 10^−3^), and dapsone-induced DRESS (OR = 40.50, p-value = 1.0784 × 10^−5^) in Thai population. The frequency of HLA-B*13:01 was found in 81.25% of dapsone-induced SCARs, 10.0% of tolerant controls and 11.49% of general Thai population. The HLA-B*13:01 has a sensitivity of 76.47% and a specificity of 92.31% for predicted dapsone-induced SCARs with the prevalence of dapsone hypersensitivity syndrome was 1.4% (2). Previous study, we found the incidence of DHS among non-leprosy patients (1.66%) was compatible to that observed among leprosy patients (1.0%) (2). Meanwhile, HLA-B*13:01 allele sensitively and specifically predicted DHS in Han Chinese leprosy patients (85.5 and 85.7%, respectively). Furthermore, DHS in Han Chinese leprosy patients were found to carry HLA-B*13:01 (OR 122.1, p-value = 6.038 × 10^−12^ and OR 20.53, p-value = 6.84 × 10^−25^), Indonesian leprosy patients (OR 233.46, p-value = 7.11 × 10^−9^), and Korean patients (OR 73.67) (25, 26, 36, 37). When we used corrected *p*-values for multiple comparison, the only HLA-B*13:01 has a statistically significant association when compared between dapsone-induced SCARs and tolerant controls and general Thai population and significantly reduce the incidence of DHS in the Chinese population (38). Moreover, the risk of dapsone-induced SCARs was significantly associated with Asian patients (Thais and Taiwanese) with the HLA-B*13:01 allele, with an OR of 36.00, 95% CI = 8.67–149.52, and *P*c-value = 2.8068 × 10^−7^. Thus, HLA-B*13:01 is strongly associated with dapsone-induced SCARs including of SJS-TEN and DRESS in leprosy and non-leprosy Asian patients. The allele frequency of HLA-B*13:01 distribution was 2–20% of Chinese, 28% of Papuans and Australian aborigines, 1–12% of Indians, 18.2% of Turkey, 8.72% of Korean, 2–4% of Southeast Asians, 1.5% of Japanese, 5.60% in Taiwanese, 5.96% of Thais, and 0% of Europeans and Africans (2, 39–41) (http://www.allelefrequencies.net/hla6006a.asp?hla_population=2842). Certainly, HLA-B*13:01 with dapsone-induced SCARs (SJS-TEN and DRESS) was strongly associated of ethnic-specific genetic in different populations. Correspondingly, HLA-B*15:02 and HLA-A*31:01 have been identified as predictive genetic markers for carbamazepine hypersensitivity in Asian and European patients (42). The biogeographical ancestry has important role in express a range of pharmacogenetics alleles and several type of SCARs. Further studies should investigate the association of pharmacogenetics marker and dapsone-induced SCARs in other population, especially Europeans and Africans.

We observed a significant association between *HLA* alleles such as HLA-A*24:07, HLA-C*03:04, HLA-DRB1*15:01, and HLA-DQB1*06:01 and dapsone-induced SCARs. The HLA-DRB1*15:01 allele was significantly associated with dapsone-induced SJS-TEN, whereas HLA-C*03:04 and HLA-DQB1*06:01 were significantly associated with dapsone-induced DRESS (*p*-value <0.05). Previous genome-wide association study had reported the association between HLA-C*03:04 and DHS in Han Chinese leprosy patients with OR = 9.00 and *p*-value = 2.23 × 10^−19^ (26). In the present study, we also found association between HLA-C*03:04 and dapsone-induced SCARs (OR = 9.00, p-value = 0.0023), SJS-TEN (OR = 13.50, p-value = 0.0212), and DRESS (OR= 7.50, p-value = 0.0155). The distribution of HLA-C*03:04 allele has been reported in different populations such as 4.37% in African Americans, 7.27% in Hispanics, 8.11% in Caucasians, 11.23% in North Americans, 10.03% in Asians, 13.70% in Japanese, 12.20% in Taiwanese, 8.09% in Thais, and 9.90% in Han Chinese (41, 43) (http://www.allelefrequencies.net/hla6006a.asp?hla_population=2842). This possibly suggests that HLA-C*03:04 allele might be a pharmacogenetics marker for dapsone-induced SCARs in many populations. Frequencies of several HLA haplotypes such as HLA-B*13:01–C*03:04, HLA-B*13:01–DRB1*15:01, and HLA-B*13:01–DQB1*06:01 were higher in dapsone-induced SCARs group compared to dapsone-tolerant controls and general Thai population. When the p-values were adjusted for multiple comparisons, associations were lost except HLA-B*13:01–C*03:04 haplotype in dapsone-induced SCARs. Nonetheless, individual HLA-B*13:01 genotypes had a high risk for dapsone-induced SCARs (SJS-TEN and DRESS) when compared with haplotypes. Although, in this study was found all dapsone-induced SJS-TEN patients without severe ocular complications (SOC), HLA-A*02:06 and HLA-B*44:03 were strong risk factor of cold medicine-induced SJS-TEN with SOC in Japanese population (31). With the presence of these alleles, further study should be conducted on these *HLA* alleles and culprit drugs-induced SJS-TEN with SOC in Thai population.

The sulfonamide structure is the basis of many drugs. Base on the sulfonamides structure can be divided into three types, consisting of sulfonylarylamines, non-sulfonylarylamines, and sulfonamide moiety-containing drugs (44). Consequently, the cross-reactivity of sulfonamide hypersensitivity reactions have been reported among sulfonylarylamines (antimicrobial sulfonamides) (45). Co-trimoxazole (sulfamethoxazole, SMX: trimethoprim, TMP) is commonly used for antibiotic, *Pneumocystis jiroveci* pneumonia (PJP) for HIV prophylaxis, organ transplantation, and cancer chemotherapy. Nevertheless, co-trimoxazole has been reported as the most common culprit drug for SJS/TEN in several countries and Thailand (46, 47). According to the data from the spontaneous reports during 1984 to 2014 by the Health Product and Vigilance Center of Thailand, co-trimoxazole is the most common culprit drug causing SJS and TEN, whereas dapsone is the 20^th^ ranked culprit drug who suffered from SJS and TEN in Thailand (http://thaihpvc.moph.go.th/thaihvc/Public/News/uploads/hpvc_5_13_0_100526.pdf). Particularly, structure of dapsone is comprised of the simplest of the sulfones, there is considerable cross-reactivity among various sulfonamide structure. The previous study showed a significant association of HLA-B*15:02, HLA-C*06:02, and HLA-C*08:01 alleles with co-trimoxazole-induced SJS-TEN in Thai patients (48). The HLA-B*15:02 allele was strongly associated with co-trimoxazole-induced SJS-TEN in Thai patients with (OR = 3.91, *p*-value = 0.0037). However, our results from this study were not consistent with the results of co-trimoxazole-induced SJS-TEN regarding the HLA-B*15:02 allele, although the frequency of HLA-B*15:02 allele in Thai population and Han Chinese is approximately 10–20% (49). Recent studies from meta-analysis and molecular dynamic simulation between HLA-B*13:01 and dapsone structure proposed that dapsone would fit within the structure of the antigen-recognition site and may change the self-peptides that bind to HLA-B*13:01 causing dapsone hypersensitivity syndrome (50, 51). The association of HLA-B*15:02 or HLA-B*13:01 alleles with cross-reactivity between sulfonamide structure and different types of SCARs needs further exploration.

In addition to the HLA alleles, drug-metabolizing enzymes have been found to play a role in the pathogenesis of SCARs. The genetic variants of cytochrome P450 (*CYP2C9*), encoding an enzyme responsible for metabolic clearance of phenytoin are strongly associated with phenytoin-induced SCARs in Taiwanese, Japanese, and Malaysians (23). CYP2C9*3 was significantly associated with phenytoin-induced SJS/TEN (OR: 4.30; 95% CI: 1.41–13.09 and p-value = 0.0133) in Thais (24). Dapsone is metabolized in the liver by nitrogen (N)-acetylation and N- hydroxylation. The N-hydroxylation is mediated by cytochrome P450 (*CYP2E1*, *CYP2C9*, *CYP2C19*, and *CYP3A4*) (29). N-hydroxylated metabolites consist of DDS-NHOH and monoacetyl dapsone hydroxylamine (MADDS-NHOH). DDS-NHOH is responsible for fever, rash, and internal organ involvement in dapsone hypersensitivity reactions (52). *CYP2C9* extensively metabolizes co-trimoxazole and influences reactive metabolites induced cytotoxicity (53, 54). In this study, we did not find the significant association between genotypes and phenotypes of *CYP2C9*, *CYP2C19*, and *CYP3A4 v*ariants and dapsone-induced SCARs (SJS-TEN and DRESS). There is an association of mucosal involvement, hepatitis, higher age, and disease occurrence with a higher risk of fatal outcome of dapsone hypersensitivity syndrome (55). Our results suggest that the severity of internal organ involvement (hepatitis) and hematological abnormalities may correlate with dapsone-induced SCARs, but dapsone dosage does not seem to affect the incidence of dapsone-induced SCARs (SJS-TEN and DRESS) in the Thai population. Nevertheless, the number of subjects in this study may not be sufficient enough to confirm all the assumptions. Further studies using a large number of samples are required for better comprehension.

In previous study, the detection of HLA‐B*13:01‐restricted dapsone and metabolite form‐responsive CD8+ clones indicates that dapsone hypersensitivity syndrome should be used as an example to discover the structural features of drug, HLA binding and interaction (56). The *in silico* model suggested that the 5-carboxamide group of CBZ might interact with Arg 62 of B pocket of HLA-B*15:02 (binding energy -37.104 kcal/mol) and Asn 63 contributes to the specificity in HLA recognition (57). In this study, we found three amino acid residues on an extra deep sub-pocket on F pocket within the antigen-binding site of HLA-B*13:01 and binding affinity of dapsone and DDS-NHOH for HLA-B*13:01 was much greater than HLA-B*13:02. Additionally, a docking model between dapsone and DDS-NHOH and HLA-B*13:01 allele was found to be appropriate because specific interaction triggers structural changes in the antigen-recognition site, allowing the protein to recognize peptides that are conformationally altered. Specific HLA allele plays a major immunopathogenesis role of drug hypersensitivity reactions, several hypotheses have been proposed to explain the interaction of HLA, drugs, peptides, and T cell (58). In brief, the hapten/prohapten model proposes that a chemically active drug or its metabolite forms a covalent bond with an endogenous peptide and then is intracellularly processed and presented by the particular HLA. While, the direct pharmacological interaction (p-i) model involves a non-covalent and labile interaction of the drug with HLA at the cell surface independent of antigen processing or T cell receptor. Another hypothesis, the altered peptide repertoire model, suggests the drug or its metabolites can bind non-covalent within the pocket of binding groove of certain HLA allele (34, 58). Thus, the altered peptide repertoire model involves the binding of dapsone and DDS-NHOH to HLA-B*13:01 allele and explains why the specific HLA-B*13:01 allele is a marker of dapsone-induced SCARs, despite the *cytochrome P450* gene is responsible for the metabolism of dapsone to dapsone hydroxylamine.

This study confirms the specific association between HLA-B*13:01 and dapsone-induced SCARs including SJS-TEN and DRESS in the Thai and Taiwanese population. Although HLA-A*24:07, HLA-C*03:04, HLA-DRB1*15:01, and HLA-DQB1*06:01 were associated with dapsone-induced SCARs, none of these associations were considered statistically significant after Bonferroni’s correction. Furthermore, there was no association between genetic polymorphisms of *CYP2C9*, *CYP2C19*, and *CYP3A4* and dapsone-induced SCARs. In addition to the specific interaction of dapsone and DDS-NHOH at the extra deep sub-pocket around the F pocket on HLA-B*13:01 allele, resulting in a change in the structure of antigen-recognition site of HLA-B*13:01 may induce altered peptides that bind to this HLA allele. Consequently, only HLA-B*13:01 might serve as a pharmacogenetics marker for screening before initiating the therapy with dapsone for the prevention of dapsone-induced SCARs.

## Data Availability Statement

The datasets presented in this study can be found in online repositories. The names of the repository/repositories and accession number(s) can be found in the article/supplementary material.

## Ethics Statement

The studies involving human participants were reviewed and approved by the ethics committee of Ramathibodi Hospital (MURA2016/105), Khon Kaen University (HE510837) and Udon Thani Hospital (22/2563). The patients/participants provided their written informed consent to participate in this study.

## Author Contributions

All authors helped to perform the research. PS’s contribution included sample collection, manuscript writing, drafting conception and design, performing procedures, and data analysis. JP, PR, JK, NN, TRu, PK, NS, AM, WA, UK, TT, KW, PJ, NK, TJ, TRe, C-WW, DN, WT, Ma, TRo, MP, and W-HC contributed to sample collection, data analysis and contribution to writing the manuscript. CS contributed to drafting conception, design, and contribution to writing the manuscript. All authors contributed to the article and approved the submitted version.

## Conflict of Interest

The authors declare that the research was conducted in the absence of any commercial or financial relationships that could be construed as a potential conflict of interest.

The reviewer TB declared a past collaboration with the author W-HC to the handling editor.
